# DSKO: Dancing through DFTB Parametrization

**DOI:** 10.1021/acs.jctc.6c00121

**Published:** 2026-05-01

**Authors:** Artem Samtsevych, Yihua Song, Tammo van der Heide, Balint Aradi, Benjamin Hourahine, Reinhard J. Maurer, Karsten Reuter, Christoph Scheurer, Chiara Panosetti

**Affiliations:** † Fritz Haber Institute of the Max Planck Society, 14195 Berlin, Germany; ‡ Bremen Center for Computational Materials Science, 9168 University of Bremen, 28359 Bremen, Germany; § Department of Physics, SUPA, 3527 University of Strathclyde, Glasgow G4 0NG, United Kingdom; ∥ Department of Chemistry, University of Warwick, Coventry CV4 7AL, United Kingdom; ⊥ Faculty of Physics, 27258 University of Vienna, 1090 Vienna, Austria; # Department of Physics, University of Warwick, Coventry CV4 7AL, United Kingdom

## Abstract

Density functional tight binding (DFTB) offers a computationally efficient alternative to ab initio methods, bridging the accuracy of density functional theory (DFT) and the speed of semiempirical models. The approximate nature of DFTB makes its reliability highly dependent on parameter quality. While recent advancements have significantly improved the parametrization of the so-called repulsive potential, the parametrization of the electronic part of the DFTB interaction remains relatively simplistic and underdeveloped. We present DFTB Slater–Koster Optimizer (DSKO), a novel framework that aims at producing accurate and transferable electronic parameter sets under rigorous physical constraints. Incorporating robust optimization algorithms and physics-informed loss functions, DSKO generates DFTB electronic parameters that yield electronic properties, such as density of states and band structures, closely matching DFT reference data. The versatility of DSKO facilitates the wide application of DFTB to materials science challenges, paving the way for routine high-fidelity semiempirical simulations.

## Graphical abstract

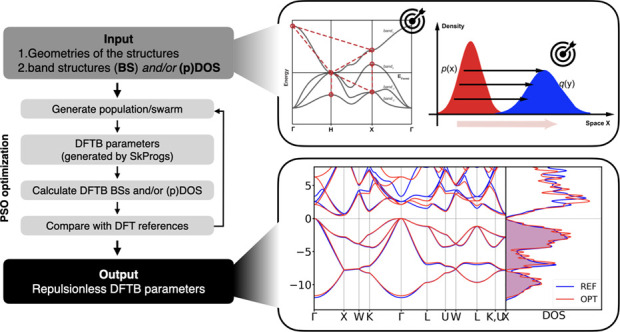

## 1 Introduction

Advances in electronic structure methods and computing power have greatly expanded the accessible size and time scales of atomistic simulations in chemistry, physics, and materials science. While density functional theory (DFT) offers a favorable balance between accuracy and efficiency, corresponding first-principles treatments still remain impractical for many scientific problems. This limitation has driven the development of semiempirical quantum chemistry methods,^1–7^ which combine DFT formalism with systematic approximations and empirical parametrization to retain essential accuracy at reduced computational cost. Among these, density functional tight binding (DFTB)^8,9^ is particularly notable for its efficiency, employing (1) a second- or third-order DFT energy expansion around a reference density obtained as a superposition of neutral confined atoms and (2) a minimal basis set of confined atomic orbitals. These approximations enable quantum mechanical treatment of systems up to a hundred million atoms.^2,10,11^ DFTB has proven effective in diverse areas, including biomolecular simulations,^12–15^ catalysis,^16–19^ and materials design.^20–26^

Despite the emergence of machine-learning interatomic potentials (MLIPs), DFTB remains attractive because it is derived explicitly from Kohn–Sham DFT.^27^ MLIPs, by contrast, reproduce the consequences of quantum behavior present in their training data, but remain effective classical models rather than quantum Hamiltonians.

The self-consistent charge formulation of DFTB naturally incorporates long-range electrostatic interactions,^28,29^ a feature that demands careful consideration in pure machine learning (ML) approaches. While recent MLIPs have made progress in incorporating charge transfer and polarization effects,^30–33^ these implementations often require complex neural network architectures and extensive training data. In contrast, DFTB’s treatment of electrostatics emerges naturally from its quantum mechanical foundation. These inherent capabilities, combined with DFTB’s interpretability and transferability, justify its continued development despite its higher computational cost compared to MLIPs.

Nevertheless, DFTB has intrinsic limitations (inherited from its DFT foundation) in describing specific quantum mechanical phenomena, particularly for strongly correlated systems, transition metal complexes, and excited states. Although recent advances have improved its electronic structure description^17,34–36^ and extended its applicability to time-dependent phenomena,^37–39^ the accuracy and transferability of DFTB ultimately depend on its underlying parametrization. In contrast to MLIPs, however, an entire set of ground- and excited-state observables can be accessed from the DFTB parametrization at essentially negligible additional computational cost, rather than requiring separate bespoke models for each quantity.

The following sections briefly summarize both the theoretical foundations and practical considerations of DFTB parameter development, with particular focus on the parametrization of the so-called electronic part. Notably, the emergence of MLIPs has created a new demand for robust electronic parameter sets for DFTB. Using electronic parameters alone, single-point DFTB calculation provides rapid, low-cost access to electronic structure information, while faster energetics for sampling are supplied by MLIPs. This new application scenario has renewed interest in the separate electronic parametrization of DFTB. Compared to the so-called repulsive part and its parametrization, electronic parametrization remains less systematically developed: most existing tools focus primarily on the repulsive potential or employ all-together strategies, leaving the electronic part limited (detailed in Section 2.3). For these reasons, we introduce the DFTB Slater–Koster Optimization (DSKO) framework, a semiautomated workflow designed to address key challenges in DFTB electronic parametrization and to facilitate systematic parameter optimization across diverse chemical environments. For readers interested in a more detailed mathematical discussion of the DFTB method and its origins, we recommend consulting key publications and recent reviews in the field.^40–42^

## 2 The Nuts and Bolts of DFTB Parametrization

DFTB is derived from Kohn–Sham DFT^27^ by expanding the total energy functional in a Taylor series around a reference electron density *n*_0_(**r**). This is directly justified by the Foulkes–Haydock interpretation of empirical tight binding as a stationary approximation to DFT.^43^ The reference electron density, typically constructed from neutral atomic densities, serves as the baseline for approximating the ground state density *n*(**r**) = *n*_0_(**r**) + δ*n*(**r**). The DFTB energy functional *E*_tot_ is thus expressed as

$$E_{\mathrm{tot}}[n_{0},\delta n]=\sum_{a}f_{a}\left\langle \psi_{a}\left|-\frac{1}{2}\nabla^{2}+V_{\mathrm{ext}}+V_{H}[n_{0}]+V_{\mathrm{xc}}[n_{0}]\right|\psi_{a}\right\rangle +\frac{1}{2}\iint \left(\frac{\delta^{2}E_{\mathrm{xc}}[n_{0}]}{\delta n\delta n^{\prime }}+\frac{1}{∥r-r^{\prime }∥}\right)\delta n\delta n^{\prime }\;dr\;dr^{\prime }-\int \left(\frac{1}{2}V_{H}[n_{0}]+V_{\mathrm{xc}}[n_{0}]\right)n_{0}(r)\;dr+E_{\mathrm{xc}}[n_{0}]+E_{\mathrm{NN}}$$ 1

where *f*_*a*_ are orbital occupation numbers, *V*_H_, *V*_xc_ and *V*_ext_ are the Hartree, exchange–correlation potentials and external interaction (including electron–ion interactions), respectively, *E*_xc_ is the exchange–correlation functional, and *E*_NN_ is the nucleus–nucleus repulsion energy.

Kohn–Sham orbitals, |ψ_*a*_⟩, entering the first term of eq 1 are expanded into atomic orbitals (AOs), |ϕ_ν_⟩, most commonly within a minimal basis set, including usually only valence orbitals. The AOs are obtained from DFT calculations for spherically symmetric, spin-unpolarized neutral atoms under an applied confinement potential *V*_conf_ which mimics the contraction of electron densities upon chemical bonding. The compressed AOs are used to construct Hamiltonian and overlap integrals of the form

$$\begin{array}{rll} H_{\mu \nu }^{0} & =\left\langle \phi_{\mu }|-\frac{1}{2}\nabla^{2}+V_{\mathrm{KS}}^{\left(2c\right)}|\phi_{\nu }\right\rangle & \\ S_{\mu \nu }^{0} & =\left\langle \phi_{\mu }|\phi_{\nu }\right\rangle & \end{array}$$ 2

where *V*_KS_^(2*c*)^ is an effective two-center Kohn–Sham potential, neglecting all crystal-field- and three-center-like integrals.^44^ Notably, this formulation applies to Hamiltonian matrix elements when the orbitals μ and ν are located on different atoms. For orbitals centered on the same atom *A*, the matrix element simplifies to the onsite energy, i.e., $H_{\mu_{A},\nu_{A}}^{0}=\varepsilon_{\mu }\delta_{\mu \nu }$ , where ε_μ_ is the eigenvalue of the atomic orbital μ. These matrix elements are precomputed via Slater–Koster transformations,^45^ and efficiently evaluated at runtime for arbitrary geometries. Within these approximations, the first term in eq 1, historically referred to as the band structure energy *E*_BS_, is directly expressed as

$$E_{\mathrm{BS}}=\sum_{a}\sum_{A,B}\sum_{\begin{array}{c} \mu \in A,\\ \nu \in B \end{array}}c_{a\mu }^{*}c_{a\nu }H_{\mu \nu }^{0}$$ 3

where the sum over *a* runs over all occupied eigenstates, and the sum over *A*, *B* runs over all pairs of atoms in the system. The diagonal elements $H_{\mu \mu }^{0}$ are equal to the eigenvalues ε_μ_ of the corresponding atomic orbital μ (often called the onsite energy). This expression forms the basis of the nonself-consistent DFTB approach, also called DFTB1, which is effectively equivalent to the Harris–Foulkes functional^43,46^ with *n*_0_(**r**) in place of the true density *n*(**r**).

The effective potential *V*_KS_^(2*c*)^ can be written by either superposition of the atomic potentials or the potential evaluated from superposed atomic densities, respectively:

$$\begin{array}{rll} V_{\mathrm{KS}}^{\left(2c\right)} & =V_{\mathrm{eff}}[n_{0}^{A}(r-R_{A})]+V_{\mathrm{eff}}[n_{0}^{B}(r-R_{B})] & \\ V_{\mathrm{KS}}^{\left(2c\right)} & =V_{\mathrm{eff}}[n_{0}^{A}(r-R_{A})+n_{0}^{B}(r-R_{B})] & \end{array}$$ 4

Density superposition constructs the total electron density as a sum of atomic densities. However, as highlighted by Seifert, this approach does not inherently lead to a more physically consistent description, particularly in DFTB1.^47^ In contrast, potential superposition allows flexible tuning of the confinement potential, providing a framework that is more consistent with the Harris–Foulkes stationary approximation.^47^ This approach, which combines atomic potentials, not only offers a simpler parametrization but has also been shown to yield more accurate band structures in some cases,^48^ though it may introduce discrepancies in representing electron–electron interactions. The choice between density and potential superposition is often dictated by the parametrization process itself, depending on which approach works best for a given system. Regardless of the strategy, it has to be noted that the atomic densities in eq 4 are compressed densities, following the same logic introduced in the confinement of the atomic orbitals to mimic the compression upon chemical bonding, thereby enabling separate compression choices for densities during parametrization. Generally, the atomic orbitals and the atomic densities are compressed at different degrees from one another (as a rule of thumb, the densities are typically compressed to a lesser degree).

The second term in eq 1 represents the Coulomb interaction corresponding to the second-order contribution of the expansion in δ*n*. In classic DFTB models, the charge fluctuations introduce a dependence of the energy on Kohn–Sham orbitals, necessitating self-consistent solutions. As such, the DFTB flavors using this expression are called Self-Consistent-Charge DFTB (SCC-DFTB) or DFTB2, and the corresponding energy term is often referred to as the self-consistent energy *E*_SCC_. Approximating it as a sum of pairwise interactions between Mulliken point charges, this can be recast into:

$$E_{\mathrm{SCC}}=\frac{1}{2}\underset{A,B}{\sum }\gamma \left(r_{AB}\right)q_{A}q_{B}$$ 5

where γ­(*r*_*AB*_) is a screening function, *r*_*AB*_ is the distance between atoms *A* and *B*, and *q*_*A*_ and *q*_*B*_ are Mulliken charges of atoms *A* and *B*, respectively. For large distances, γ­(*r*_*AB*_) asymptotically approaches as 1/*r*_*AB*_, while it smoothly converges to the Hubbard parameter *U* as *r*_*AB*_ → 0.

This leaves as remaining term in eq 1 the sum of all the contributions to the total energy that have so far not been accounted for. These are lumped together in the so-called repulsive energy *E*_rep_. An explicit computation of this contribution has been explored before but has yielded modest accuracy.^49,50^ This is due to the fact that an explicitly calculated repulsive energy would fully recover the total DFT energy only if no approximations were made in the expression of the *E*_BS_ and *E*_SCC_ terms. Thus, the approximations introduced to these terms necessitate compensating via empirical fitting of *E*_rep_.

To recap, the expression for the DFTB2 total energy is conceptually subdivided into three terms with distinct meanings:

$$\begin{array}{rll} E_{\mathrm{tot}}[n_{0},\delta n] & =\underset{\mathrm{electronic}}{\underset{︸}{E_{\mathrm{BS}}[n_{0}]+E_{\mathrm{SCC}}[n_{0},\delta n]}}+E_{\mathrm{rep}}[n_{0}] & \\ & =E_{\mathrm{el}}+E_{\mathrm{rep}} & \end{array}$$ 6

where the *E*_BS_ and *E*_SCC_ terms are combined as electronic part *E*_el_, and *E*_rep_ represents a correction to the latter to recover *E*_tot_. The beauty of this expression is that each of these terms depends on some numerical parameters (onsite energies ε_μ_, Hubbard *U*_μ_, confinement potentials, and some suitable expression for the repulsive energy), but none depend on all of them simultaneously. As such, the electronic and repulsive contributions can be tuned independently, strongly motivating a divide-and-conquer approach to DFTB parametrization.

It is worth mentioning at this point that not all numerical parameters are created equal: a clear hierarchy exists in their degree of empirical character. Onsite energies ε_μ_ and Hubbard parameters *U*_μ_ can be calculated from first principles. If treated as tunable, they should be adjusted only if necessary because they carry explicit physical meaning. The choice of orbitals in the basis set (only valence ones or an extended basis set with virtual orbitals) and confinement potentials (both for electron density and for wave functions) are the only truly empirical objects in the electronic parametrization, and therefore are mandatorily tunable. Fortunately, they can be effectively represented with simple functional forms. The repulsive potential, in contrast, is not only a fully empirical correction, but to complicate things further, no suitable “foolproof” ansatz for its functional form exists. As such, DFTB parametrization is inherently multifaceted, leading to a wide range of approaches and techniques developed over the years (see Section 2.3 for a detailed overview).

### 2.1 Electronic Parametrization

Practically, the parametrization of the electronic energy contribution *E*_el_ comprises two fundamental stages: (i) solving the one-center problem to obtain compressed atomic densities and basis functions, and (ii) addressing the two-center problem to compute Hamiltonian and overlap matrix elements as functions of interatomic distance. Orbital-resolved confinement of wave functions, as opposed to uniform (nonresolved) confinement, allows for targeted adjustment of individual orbitals based on their bonding characteristics and thus can significantly improve the resulting electronic properties.

The form of the confinement potential *V*_conf_ for both wave function and density compression is arguably the most central aspect of electronic parametrization. In many practical scenarios, it is sufficient to optimize the confinement potentials until a certain property of choice (e.g., band structures) matches the DFT reference. As discussed above, onsite energies ε_μ_ and Hubbard *U*_μ_ values rarely necessitate tuning. If required, it should be preferentially attempted to tune only the values for unoccupied orbitals, with some caveats. First, unoccupied orbitals do not affect the total energy but only the shape and positions of (mostly conduction) bands for elemental solids, but do affect the total energy for composite solids or more generally when any charge transfer is present. Second, tuning them to achieve improved conduction bands for a certain target might affect transferability, as one solid’s conduction band can be part of another solid’s valence band. Third, and more fundamentally, eigenvalues for unoccupied orbitals are ill-defined to begin with, so an extended basis should preferentially be adopted only if strictly necessary (e.g., the correct representation of the indirect band gap in diamond silicon, see Section 4.3)

The most common form is the power-law potential, expressed as

$$V_{\mathrm{conf}}\left(r\right)={\left(\frac{r}{r_{0}}\right)}^{s}$$ 7

where *r*_0_ is an onset radius and *s* is the exponent. Quadratic confinement (*s* = 2) is particularly popular due to its simplicity, requiring only one tunable parameter (*r*_0_), which makes the parametrization process easier.^9,48,51^ However, this simplicity limits the flexibility in finely shaping the compressed orbitals and electron density in order to capture electronic properties more accurately. This flexibility is recovered by making the exponent tunable, clearly at the price of doubling the dimensionality of the search space.

As a further step ahead in flexibility, the Woods–Saxon (WS) potential,^52,53^ given by

$$V_{\mathrm{conf}}\left(r\right)=-\frac{W}{1+\exp\left(-a\left(r-r_{0}\right)\right)}$$ 8

introduces three tunable parameters: *W* (height), *a* (slope), and *r*_0_ (half-height radius), and provides greater control over the radial behavior of orbitals. Moreover, the WS potential yields correct exp­(−α*r*) asymptotic behavior of atomic orbitals, whereas the orbitals obtained with the (*r*/*r*_0_)^*s*^ potential vanish faster with exp­(−α*r* ^1+*s*/2^) asymptotics.^53^ The WS potential also minimally perturbs the atomic core region, especially for larger *a* values, where it closely resembles a square well with smooth walls. However, the increased flexibility of the WS potential comes at the cost of higher optimization complexity. The effects of power-law and WS confinement potentials on the atomic wave function are illustrated in Figure 1.

**Figure 1:**
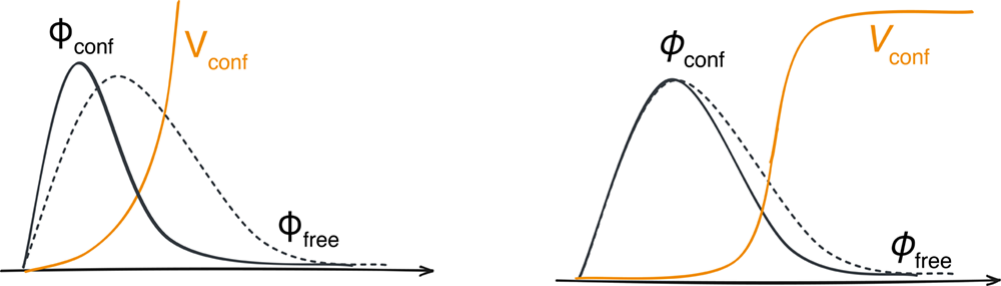
Sketches illustrating the effect of the confinement potential *V*_conf_ on a fictitious wave function ϕ_free_: power (left) and Woods–Saxon (right).

### 2.2 Repulsion Parametrization

Despite its name, *E*_rep_ can exhibit both repulsive and attractive characteristics, depending on the parametrization. While some classical formulations—such as Chadi’s original idea from the 1970s—are strictly repulsive,^54^ *E*_rep_ becomes strictly repulsive only at short interatomic distances. Crucial for modeling short-range Pauli exclusion and core–core interactions,^42^ its empirical parametrization against DFT data is preferred over explicit calculation. The most common approach approximates *E*_rep_ through pairwise interactions, summing over atom pairs with a short-ranged potential *V*_rep_(*R*_*ij*_) that vanishes beyond a cutoff radius *R*_cut_.^55^ Early representations of the repulsive potential employed linear combinations of cutoff polynomials for dimers and symmetric clusters.^40,50^ Subsequent developments explored various functional forms, with exponentials combined with polynomials and splines being most prevalent.^56–60^ Fully automated frameworks^61–63^ address the limitations of manual fitting. ML techniques, particularly Gaussian Process Regression (GPR), have emerged for fitting pairwise potentials for various systems,^64^ offering regularization and simultaneous fitting for multiple elements.

However, pairwise potentials struggle with angularly dependent forces and many-body interactions, especially in diverse bonding environments.^65^ To address these limitations, researchers have incorporated many-body effects into *E*_rep_. Goldman et al. employed the Chebyshev Interaction Model for Efficient Simulation (ChIMES) to represent two- and three-body interactions using Chebyshev polynomials, enabling efficient construction of multicenter repulsive energies.^60^ Deep Tensor Neural Networks (DTNNs) provide an even more generalized approach for modeling the nonlinear many-body repulsive energy term.^66^ A recent hybrid approach, MLTB,^67^ further extends this concept by employing a machine learning neural network potential as a many-body correction to the DFTB repulsive term, significantly improving both transferability and extensibility for complex systems.

### 2.3 Existing Tools and Packages

DFTB parametrization tools have evolved using two main strategies: divide-and-conquer, which optimizes electronic and repulsive parameters separately, and all-together, which performs simultaneous optimization. The hotbit package^40^ provided the first publicly available automated divide-and-conquer approach, offering tools for separate optimization of electronic parameters (via confinement potentials and eigenvalue matching) and repulsive parameters (via spline fitting of force differences). Van den Bossche et al. expanded this partitioned methodology through two complementary tools: Hotcent^34,68,69^ performs electronic parametrization via confined atomic DFT calculations with rigorous orbital compression control, while TANGO^70,71^ optimizes repulsive potentials through global energy and force matching across DFT-derived potential energy surfaces. SkProgs^72^ provides comprehensive utilities for diverse electronic configurations, including scalar relativistic effects via the zero-order relativistic approximation (ZORA),^73^ representing the most complete electronic parametrization generator to date. These tools (hotbit, Hotcent, and SkProgs) generate Slater–Koster files by utilizing atomic DFT calculations with Hartree–Fock-based atomic reference calculations,^74,75^ extended with DFT exchange–correlation potentials through the LibXC library.^76^ Advancements have yielded automated approaches for repulsive fitting^77,78^ and all-together tools for specific chemical species.^28,57,61,79–82^ Recent semiautomatic repulsive parametrization tools have begun to leverage machine learning and mini-batch optimization techniques for specific chemical elements.^64,83^ Extending these ideas, hybrid approaches now combine tight-binding models with machine learning to improve accuracy and transferability, as exemplified by the TBMaLT toolkit^84^ and DeePTB,^39^ which learns environment-adaptive Slater–Koster parameters from ab initio bands to enable efficient evaluation of DOS, optical, and transport properties.

Given the substantially different nature of electronic (property-oriented) and repulsive (data-oriented) parametrization, here we fully embrace the divide-and-conquer strategy. Further, we consider the problem of repulsive fitting, traditionally regarded in the community as the most difficult, essentially solved by the recent approaches presented in Section 2.2. Thus, we focus in the following exclusively on the electronic parametrization.

## 3 The DSKO Approach

Many existing approaches for generating DFTB parameters, including those discussed in Section 2.3, face several interconnected challenges. First, they mainly rely on in-house codes or ad hoc workflows (which are sometimes not publicly available), hindering reproducibility and widespread adoption of these tools. Another significant issue is the rapid growth in the complexity of the parameter space. While the number of tunable parameters for the electronic parametrization scales as O(N) with the number of chemical species, the pairwise (i.e., the simplest) form of repulsion entails quadratic scaling in parameter space complexity. This scaling intensifies further in many-body formulations of the repulsion, which exhibit higher-order combinatorial dependencies. Here, even the electronic parametrization becomes high-dimensional due to accumulating per-species parameters. For example, a minimal implementation with a quadratic confinement requires one parameter per species, while power and Woods–Saxon confinement types require two and three tunable parameters, respectively. Furthermore, orbital-resolved confinement introduces an additional factor *N*_orbitals_ per species, while further parameters arise from density compression optimization (additional 1, 2, or 3 tunable parameters per species, depending on the confinement type). Moreover, empirical fine-tuning of Hubbard *U*_μ_ values (additional *N*_orbitals_ tunable parameters per species) and onsite energies ε_μ_ (additional *N*_orbitals_ tunable parameters per species) is sometimes needed, especially to reproduce conduction bands. This parameter accumulation results in a space that becomes high-dimensional even for binary systems when pursuing a fully flexible optimization scheme, and brings concomitant challenges in finding the global minimum. Further, there is high variability in the composition of the parameter space (combination of confinements for electron density, wave functions, and values for onsite energies ε_μ_ and Hubbard *U*_μ_). The particular choice of search space is not standardized and varies for different DFTB parametrization tools. The final limitation of existing approaches lies in the choice of fitness function during optimization, which significantly influences the search process, especially in nonconvex landscapes, characterized by multiple local minima. Addressing these challenges requires efficient algorithms capable of handling the high dimensionality of the parameter space without compromising accuracy or demanding excessive computational resources. There is a clear need for more versatile and efficient strategies that can adaptively optimize DFTB parameters, as well as standardized, open-source frameworks to streamline parameter development and validation across diverse chemical systems.

In the present work, we therefore introduce DSKO (DFTB Slater–Koster Optimizer), a code which streamlines the optimization of electronic structure parameters and offers features that help preserve transferability across diverse chemical environments. The workflow begins with the input of target geometries and associated electronic properties, which serve as reference data. These properties can include band structures, total density of states (DOS) and projected DOS (pDOS), or any combination thereof. DSKO initializes parameter candidates within a chosen search space (see Section 3.1), then converts these to repulsion-less Slater–Koster tables using SkProgs.^72^ Each candidate set is used to perform DFTB calculations that generate electronic properties, which are quantitatively compared to the reference data through a multicomponent loss function, *L* (Section 3.3). Global optimization algorithms (Section 3.2) drive this process through preset iterations, refining parameters to minimize *L*. The final output delivers optimized DFTB parameters that best reproduce the target electronic properties across the input structures, as illustrated in Figure 2.

**Figure 2:**
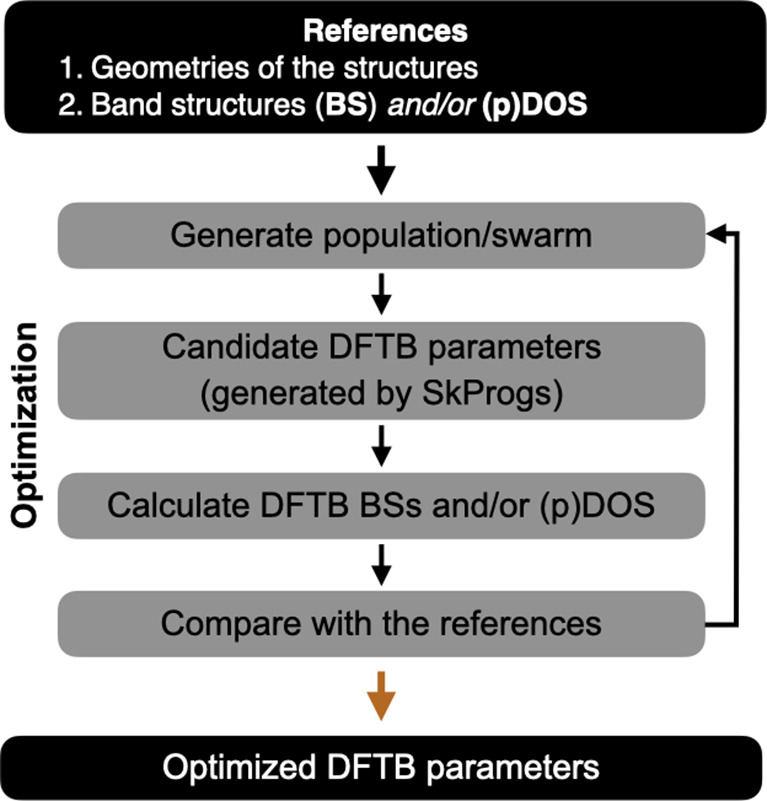
Schematic workflow of the DSKO framework for optimizing electronic DFTB parameters. The optimization process employs population-based algorithms to iteratively refine DFTB parameters over a predefined number of steps, aiming to improve agreement with the electronic properties of the reference structures.

### 3.1 Parameter Space Definition

The effectiveness of electronic parameter optimization hinges on a carefully defined and appropriately constrained parameter space. Importantly, DSKO is designed to facilitate both the de novo generation of parameter sets and the use of specific reference parameters from existing (or preobtained) parameter sets, maintaining consistency with prior parametrizations or to serve as a starting point for further optimization. This allows researchers to leverage existing knowledge while tailoring parameters to specific systems or properties of interest.

At the electronic structure level, each atomic species introduces multiple degrees of freedom through its basis set configuration and interaction parameters. For each atomic species, the choice of confinement potential *V*_conf_—applied to orbitals in the basis set (either shell-resolved or nonshell-resolved) and to density compression—determines the minimum number of tunable parameters (see Section 2.1). These parameters, together with the optional fine-tuning of onsite energies ε_μ_ and electron–electron repulsion described by the Hubbard parameter *U*_μ_, constitute the parameter search space. So, for a given optimization process, the parameter space looks as follows:

- (optional) a set of reference DFTB parameters
- for each atomic species *A*_*i*_, *i* ∈ {1,..., *N*_species_}­
  1. orbital confining parameters: [*r*_0_^*i*,μ,orb^, *a* ^*i*,μ,orb^, *W* ^*i*,μ,orb^] for Woods–Saxon confinement, or [*r*_0_^*i*,μ,orb^, *s* ^*i*,μ,orb^] for power-law confinement
    - shell-resolved orbital confining parameters (different for each orbital μ)
    - nonshell-resolved confining parameters (same values for each orbital μ)
  2. density confining parameters: [*r*_0_^*i*,dens^, *a* ^*i*,dens^, *W* ^*i*,dens^] for Woods–Saxon confinement, or [*r*_0_^*i*,dens^, *s* ^*i*,dens^] for power-law confinement
  3. (optional) set of shell-resolved onsite energies ε_*i*,μ_ (for each orbital μ),
  4. (optional) set of shell-resolved Hubbard parameters *U*_*i*,μ_ (for each orbital μ)

where μ is the angular momentum of species *A*_*i*_.

The modular architecture of DSKO supports flexible and extensible definition of the parameter space, allowing to intuitively address the specific requirements of diverse chemical systems or properties and to gradually introduce increasing degrees of fine-tuning if needed.

### 3.2 Global Optimization Strategy

Particle Swarm Optimization (PSO)^85^ serves as the foundation for the DSKO framework, though the methodology remains optimizer-agnostic and can be coupled to alternative global optimization approaches, like Bayesian Optimization (BO).^86,87^ Previous studies, including the work of Chou et al.,^61^ have demonstrated the effectiveness of heuristic algorithms in DFTB parametrization, establishing a robust precedent for population-based optimization strategies. PSO, inspired by swarm intelligence, efficiently explores the search space by iteratively improving candidate solutions with a combination of individual and collective criteria. In contrast, BO leverages probabilistic models to guide the search process, making it particularly effective for expensive-to-evaluate objective functions. In both methods, each particle represents a single parametrization candidate generated within the search space. Their (hyper-)­parameters can be tuned to balance between exploration and exploitation, but DSKO provides sensible defaults that have been proven to be robust in high-dimensional optimization.^88,89^ Both BO and PSO have proven efficient for optimizing DFTB confinement parameters targeting ideal, low-complexity crystals and with a reduced list of tunable parameters.^61,81,90^ Within the DSKO code, we currently employ PSO via the PySwarms^91^ Python package.

### 3.3 Loss Function Design

A cornerstone of our methodology is the development of a sophisticated loss function that comprehensively assesses the quality of the generated DFTB parameter set across multiple electronic structure properties. Our approach introduces several innovative components, including so-called decoding masks for the band structure, alongside DOS similarity metrics, as well as a penalty term for deviations from existing reference DFTB parameters when modifying them. The loss function, *L*, integrates these three components and mathematically can be formulated as

$$L=w_{\mathrm{BS}}\cdot L_{\mathrm{BS}}+w_{\mathrm{DOS}}\cdot L_{\mathrm{DOS}}\left(+w_{\mathrm{ref}}\cdot L_{\mathrm{ref}}\right)$$ 9

where *L*_BS_ and *L*_DOS_ stand for the difference between the reference and DFTB band structures and (p)­DOSs, respectively. *L*_ref_ is a reference penalty term introduced to systematically control deviations from a chosen set of pre-existing DFTB parameters during optimization. This term is only activated when the parametrization process is intended to refine, update or extend an existing parameter set, thereby encouraging the optimized parameters to remain close to the reference values unless a significant improvement in the target properties is achieved. The weights {*w*_α_} are selected to make the total loss *L* unitless and to tune the relative importance of each component, enabling balanced optimization that enhances both electronic structure accuracy and fidelity to existing parameter sets. Designed for extensibility, the DSKO modular architecture readily accommodates future integration of additional terms into the loss function *L*, such as molecular eigenvalue matching or partial charge correlations.

#### 3.3.1 Band Structure Loss

A prevalent approach is to compare band structures directly by calculating the *L*1 norm of all the pointwise differences between DFT reference and DFTB bands within specified energy ranges (typically −15 to 5 eV from the Fermi level), defining a fitness function *L*_BS_. However, this approach presents two significant challenges. First, the high dimensionality of the resulting feature landscape complicates the search for the global minimum. Second, the landscape’s insensitivity to small shifts of the bands creates shallow minima, further complicating the search for a true global minimum. Though replacing point-to-point comparisons with the deviation between the DFT and DFTB distance matrices between specific points of the band structures (as done, for example, in ref 24) addresses the sensitivity issue, the dimensionality problem persists.

We address these limitations through strategic dimensionality reduction. Rather than incorporating all band structure points, we select specific points (SPs) at high-symmetry positions, constructing a reduced distance matrix, termed a decoding mask (DM), between those. The DM takes the mathematical form DM = [*d*_αβ_] where *d*_αβ_ = |ε^α^ – ε^β^| represents the energy difference between each pair of SPs (α, β), and α and β are compact identifiers representing an SP label and the band index. The root-mean-square deviation between reference and DFTB decoding masks

$$L_{\mathrm{BS}}=\mathrm{RMSD}\left({\mathrm{DM}}_{\mathrm{ref}},{\mathrm{DM}}_{\mathrm{DFTB}}\right)$$ 10

provides an intrinsic measure of band structure fidelity that is agnostic to both rigid energy shifts and occasional problematic features in the band structures, such as band degeneracies or band shapes and positions that can never be correctly reproduced by DFTB due to the limited flexibility of the basis set. Figure 3 illustrates a schematic DM representation for a fictitious, exemplary band structure. By carefully choosing which points and distances to include, this approach allows discarding of redundant or problematic information while maintaining physical interpretability through its focus on (1) symmetry-constrained critical points and (2) the relative relationship of bands.

**Figure 3:**
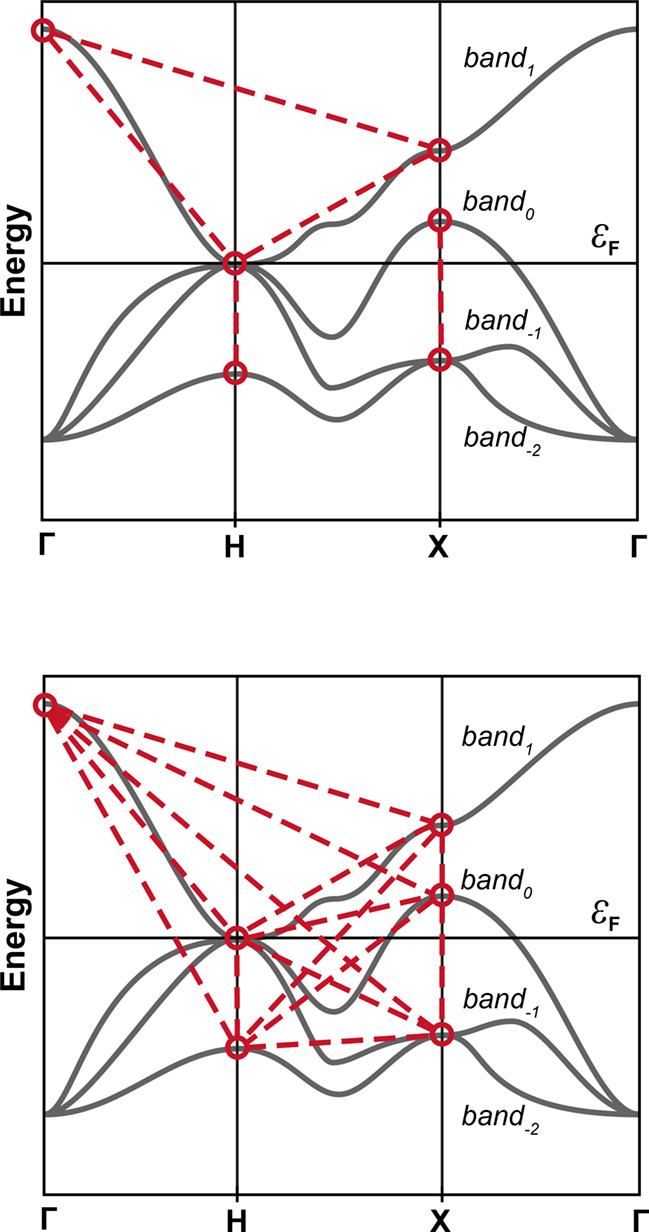
Schematic visual interpretation of the pair-by-pair (top) and set-of-points (bottom) definitions of the decoding mask (DM) for a fictitious exemplary band structure (gray lines). Red circles and dashed segments represent, respectively, the points and distances between them included in the calculation of the loss function. The pair-by-pair DM in the example can be written as [[Γ_1_, H_0_], [Γ_1_, X_1_], [H_0_, H_–2_], [H_0_, X_1_], [X_0_, X_–2_]]. The point-by-point DM can be written as {Γ: [1], H: [0, −2], X: [0, 1, −2]}.

For input specification, we adopt the following convention: we denote as band index zero the band corresponding to *N*_val_/2, where *N*_val_ is the total number of valence electrons. This corresponds to the band right below the Fermi level for insulators and semiconductors, and it is the band that straddles the Fermi level for metals. For example, Γ_0_ will then correspond to the value of that band at Γ, whereas Γ_1_ and Γ_–1_ refer to the bands above and below, and so on. Using this notation, the DM can be defined in two alternative ways: (1) pair-by-pair, as an explicit list of [SP_*i*_^*n*^, SP_*j*_^*m*^] pairs, where only the chosen distances will be included, or (2) point-by-point, as a dictionary {SP^*n*^: [*i*, *j*, *k,*...], SP^*m*^: [*p*, *q*, *r*,...],...}, where the distances for all possible pair combinations will be included. For magnetic systems, the notation is extended to account for spin channels. In list-type DMs, this is expressed as [SP_*s*,*i*_^*n*^, SP_*s*,*j*_^*m*^] for a selected pair of SPs, while dictionary-like DMs adopt the notation {SP_*s*_^*n*^: [*i*, *j*, *k*,...], SP_*s*_^*m*^: [*p*, *q*, *r*,...],...}. Here, *s* ∈ {*u*, *d*} denotes the spin channel (*u*: spin-up, *d*: spin-down), and indices *i*, *j*, *k*,... represent band indices.

We emphasize that selecting an overly simplistic DM can introduce significant errors. As illustrated by the example in Figure 4, if e.g. only intra-SP distances are chosen, the reference semiconductor may be erroneously optimized to a metal, since the corresponding distances remain almost unchanged when both bands are shifted downward at Γ. Consequently, the PSO cannot differentiate between a candidate parametrization that yields the DFTB band structure shown in Figure 4, left, and the true optimum, as both result in almost identical loss function values. By contrast, as shown in Figure 4, right, including band pairs that span distinct SPs prevents such “accidentally correct” solutions, thereby enhancing the robustness of the optimization, eliminating spurious minima, and preserving the physical semiconducting nature of the target.

**Figure 4:**
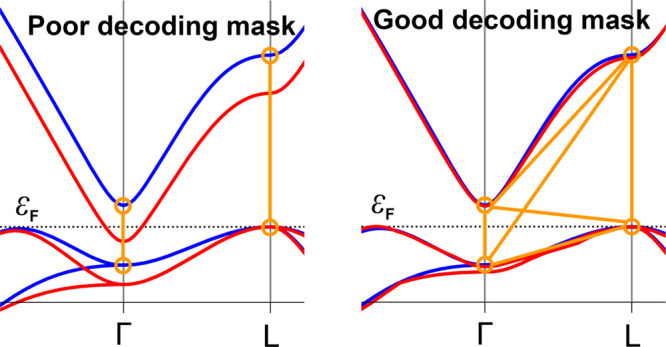
Contrasting outcomes of decoding mask strategies in DSKO parametrization. The left panel demonstrates how intra-SP band pair selection without further constraints is insufficient to correctly drive the PSO to the physically correct minimum. The interband distance for a wrong candidate solution (red) is not far from the target distance at both Γ and L (orange segments), thus not affecting the loss function. As a result, this choice of DM produces a spurious minimum in the loss function, and the resulting solution is artificially metallic rather than semiconducting like the reference (blue). The right panel illustrates how including inter-SP pairs (especially Γ_1_ to L_0_) introduces enough additional constraints to enforce the semiconducting character of candidate solutions.

To reduce the amount of manual trial-and-error in constructing DMs, we additionally provide an interactive web-based companion tool that visualizes reference band structures and allows users to select the corresponding SPs for the DM. A brief description and access details for this tool are given in Data Availability Statement Section.

#### 3.3.2 Density of States Loss

The DOS encapsulates rich electronic structure information in a condensed form, offering a natural dimensionality reduction compared to band structure-based approaches, while simultaneously capturing both energetic and state distribution information. The total DOS *D*(*E*) can be formally expressed as a sum over *N*_es_ energy states (both occupied and unoccupied) of the system:

$$D(E)=\sum_{i=1}^{N_{\mathrm{es}}}\delta (E-\varepsilon_{i})$$ 11

where, in practical calculations, Gaussian broadening is typically selected as the smearing function δ. However, alternative schemes (e.g., Fermi–Dirac, Marzari–Vanderbilt and Methfessel–Paxton) may be employed depending on the specific requirements of the calculation.

Beyond the total DOS, users may also employ the pDOSs as a reference, which resolves the total DOS onto specific atomic orbitals or atomic sites. This approach allows for a more nuanced comparison, capturing the chemical and orbital character of the electronic states.

Recent advances in ML for DOS-targeted optimization^92,93^ have demonstrated that both DOS and pDOSs can be employed as quantitative descriptors. To numerically assess the similarity between reference and candidate (p)­DOS distributions, we use the Wasserstein distance, also known as Earth Mover’s Distance:^94^

$$W(D_{i},D_{j})=\underset{\gamma \in \Gamma (D_{i},D_{j})}{\inf}\;\int_{R^{2}}|E-E^{\prime }|\;d\gamma (E,E^{\prime })$$ 12

where Γ­(*D*_*i*_, *D*_*j*_) is the set of all joint distributions with marginals *D*_*i*_(*E*) and *D*_*j*_(*E′*), and |*E* – *E′*| represents the cost of transporting spectral weight from energy *E* in *D*_*i*_ to energy *E′* in *D*_*j*_. The Wasserstein distance provides a physically intuitive metric for comparing DOS distributions, as it quantifies the minimal amount of work required to transform one distribution into another. Being equivalent to the *L*_2_ distance between inverse integrated DOS functions, it ensures consistent and interpretable comparisons of electronic structure.

#### 3.3.3 Reference Parameter Loss

The deviation of fitted parameters from existing reference values (if present), is simply expressed as

$$L_{\mathrm{ref}}=\underset{j}{\sum }w_{\mathrm{ref},j}{\left(P_{j}^{\mathrm{cand}}-P_{j}^{\mathrm{ref}}\right)}^{2}$$ 13

where *P*_*j*_^cand^ denotes a (sub)­set of candidate parameters as presented in Section 3.1, *P*_*j*_^ref^ represents its corresponding set of reference values, and *w*_ref,*j*_ are additional weights assigned to individual tunable parameters in the search space, enabling selective fidelity to reference values as required by the target system. This formulation incorporates prior parametrization data when extending parameter sets to new chemical species or system classes, allowing adjustable flexibility. For de novo generation of parameter sets, all *w*_ref,*j*_ are set to zero i.e., no reference bias is applied. Notably, this loss function term is particularly useful in the parametrization of multicomponent systems, where the dimensionality of the search space can grow quickly. PSO is known to perform well in relatively high-dimensional spaces (up to around 30 dimensions^95^), but it can struggle to converge beyond that regime. While increasing the particle swarm size can alleviate this issue, we rather recommend, when targeting multicomponent systems, a hierarchical strategy: first, run separate PSO for pristine and binary combinations, and only at a second stage target multicomponent structures, exploiting this loss function term with the previously obtained parameters as references.

### 3.4 Additional Optimization Features

For spin-polarized systems, the optimization protocol simultaneously treats both spin channels within the DFTB formalism,^44,96^ ensuring accurate magnetic moments and spin-dependent properties. This is practically implemented as setting DMs for both spin channels.

The inclusion of extended valence configurations addresses limitations of the minimal basis set by incorporating higher-lying virtual orbitals, which are essential for capturing conduction bands and hybridization effects, albeit at the cost of increased parameter space complexity.

For elements with multiple accessible electronic configurations, such as transition metals, parameter sets must describe various oxidation states and coordination environments. This is achieved by specifying multiple target band structures, typically employing an extended basis set with virtual orbitals. As a result, including diverse reference structures in the optimization with additional input parameters potentially yields a compromise that balances accuracy across different states.^97^

## 4 Electronic Parametrization in Practice

To illustrate the performance of the DSKO optimization procedure, we selected several representative systems: lithium, nickel, silicon, and silica. These systems were chosen for their technological relevance, diverse electronic properties, and most importantly, to systematically demonstrate key aspects of electronic parametrization across fundamentally different material types. The selected examples present increasing complexity: lithium is a simple metal, nickel is a magnetic transition metal, silicon is a semiconductor with an indirect band gap, and silica is a binary compound. Complete information on the computational setups (DFT parameters for reference band structures, DFTB input parameters and optimizer configuration) are provided in the Supporting Information, Section 1.

### 4.1 Lithium

We start with presenting the results of our DFTB parametrization for lithium and its validation across different crystal structures. The parametrization was performed using an extended basis set of [2s, 2p] for Li atom. Lithium is one of those cases for which restricting the basis set to occupied valence orbitals does not correctly reproduce the band structure (see e.g., ref 24). The optimization of DFTB parameters involved searching within the space of eigenenergies for each orbital of a free Li atom, shell-resolved wave function confinement and density confinement (both confinements have Woods–Saxon form) using potential superposition.

The body-centered cubic (bcc) equilibrium phase of lithium (mp-51 from Materials Project (MP) database) and its compressed form with a compression factor of 0.9 served as our reference structures for parameter fitting. The pair-to-pair DM included distances between the Γ_0_ and some points from higher bands as represented by the list [[Γ_0_, Γ_1_], [Γ_0_, H_0_], [Γ_0_, N_0_], [Γ_0_, N_1_], [Γ_0_, P_0_], [Γ_0_, P_3_]] and illustrated in Figure S1. For the compressed structure, the distance [Γ_0_ – Γ_1_] was excluded after observing, in a preliminary PSO run (shown in Figure S1), that the Γ_1_ point is disproportionally shifted upward in DFTB, while it is well-placed in the equilibrium band structure; this system-specific adjustment improved the agreement between DFTB and the PBE reference and is not a routine or automated feature of the DSKO workflow. As such, including it would drive the PSO toward wanting to minimize a large singular deviation in the RMSD between the decoding masks, throwing the optimization off for the sake of an unimportant, high-energy feature. This is an example of how a “problematic” point should be excluded from the decoding mask. An additional, targeted decoding mask was added to better reproduce the curvature at the Γ point for the equilibrium structure, including distances between Γ_0_ and Γ_1_, H_0_, P_0_.

Details of the optimized parameters are provided in the Table S1. As one can see in Figure 5, the optimized parameters reproduce the bcc lithium accurately, capturing the metallic character of the band structure, particularly the band dispersion near the Fermi level. To assess the transferability of our parameters, we performed validation calculations on hexagonal *P*6_3_/*mmc* and trigonal *R*3̅*m* lithium polymorphs (mp-10173 and mp-1018134 from the MP database, respectively).

**Figure 5:**
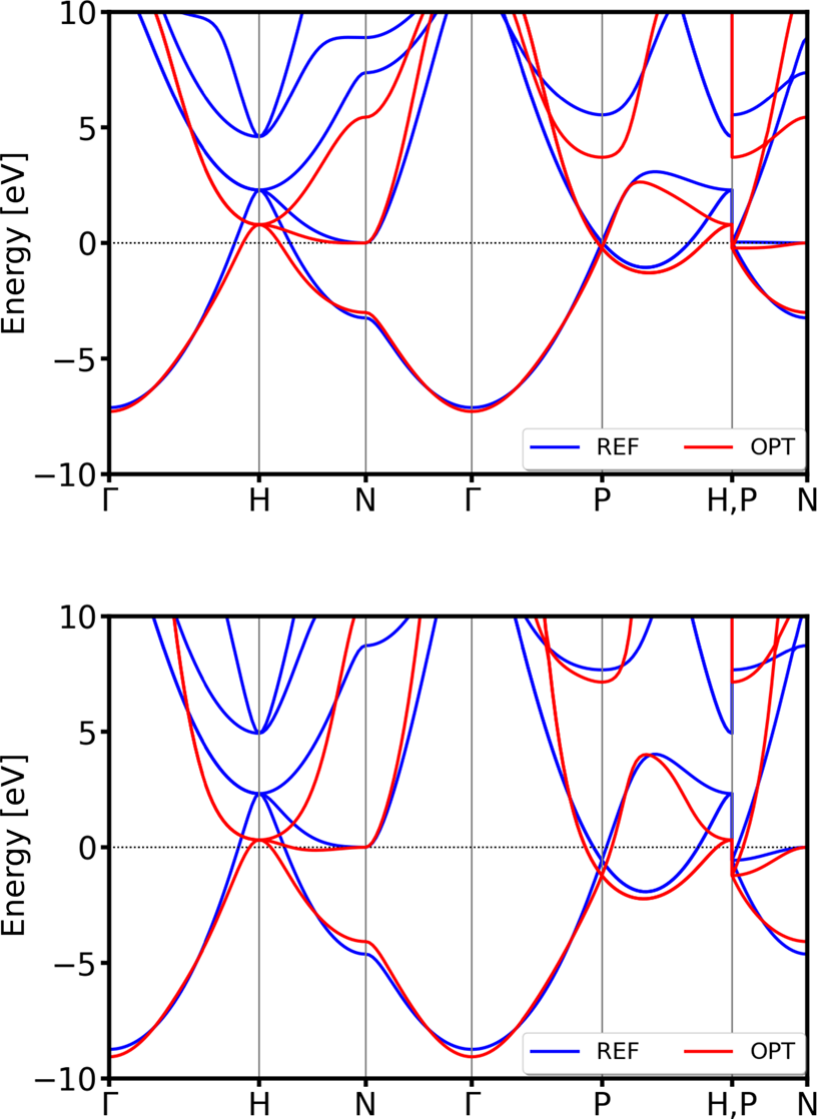
DSKO-optimized band structure (red lines) vs the DFT-calculated reference band structure for body-centered cubic (bcc) lithium (mp-51 from the MP database) at both equilibrium geometry (top) and a compressed geometry with a compression factor of 0.9 (bottom). For visualization clarity, all bands are aligned to the first conduction band above the Fermi level at the special point N, rather than to the DFT Fermi level. This unconventional alignment is chosen solely to enhance the readability of the plots.

The results demonstrate excellent agreement with DFT for these validation structures, including an accurate representation of the trigonal phase’s complicated band features (see Figure 6).

**Figure 6:**
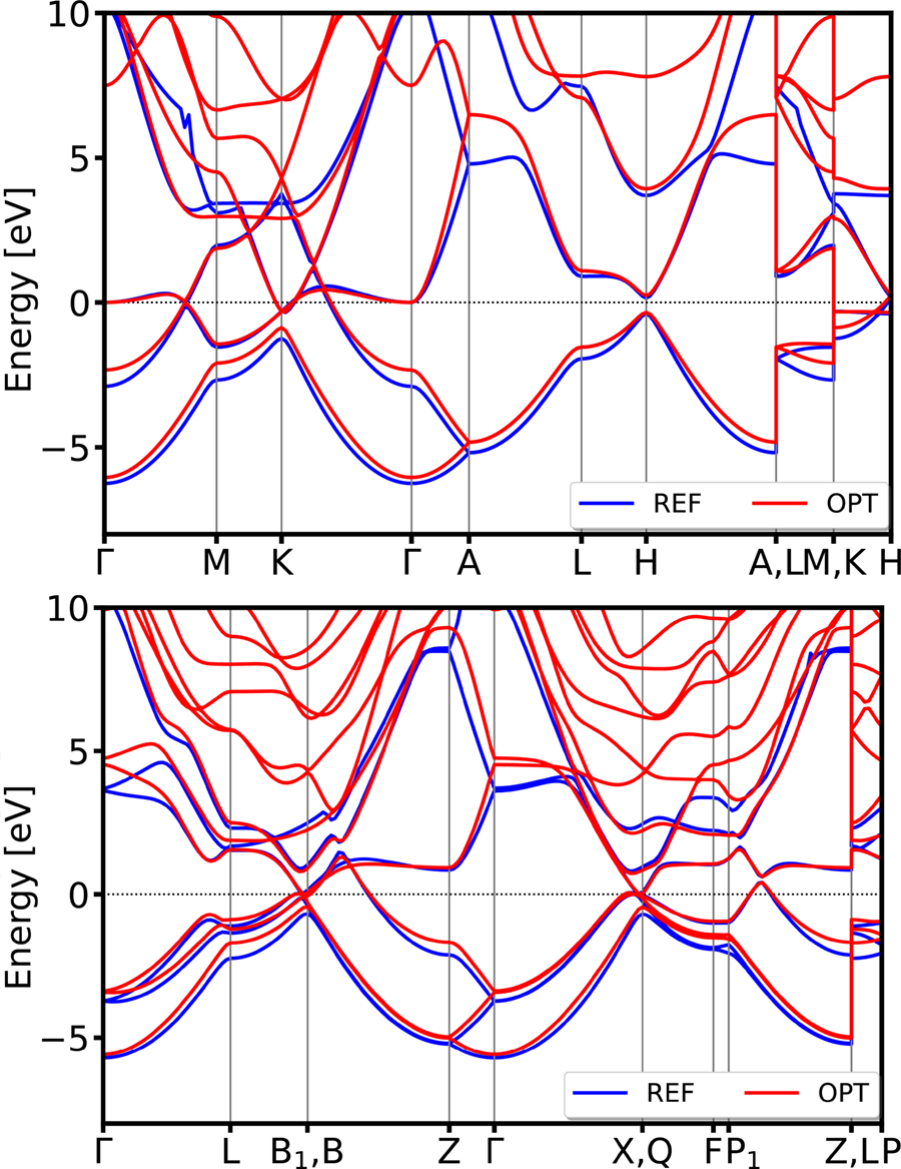
Validation of the band structures of hexagonal *P*6_3_/*mmc* (mp-10173 from the MP database) (top) and trigonal *R*3̅*m* (mp-1018134 from the MP database) (bottom) polymorphs of Li. For visualization clarity, all bands are aligned with respect to the DFT Fermi level.

### 4.2 Nickel

Next, we present the results of our DFTB parametrization for nickel and its validation across different crystal structures. The parametrization involved optimizing shell-resolved wave function confinement and density confinement, both modeled with a Woods–Saxon form, using density superposition. No tuning of eigenenergies ε_μ_ or Hubbard *U*_μ_ values was performed. An extended basis set of [3d, 4s, 4p] orbitals was used for Ni. Equilibrium simple cubic nickel (mp-23 from the MP database) was chosen as the reference structure. The DM for the input structure is the list [[Γ_0_, Γ_2_], [Γ_0_, Γ_–2_], [Γ_0_, Γ_–4_], [Γ_0_, X_1_], [X_1_, X_–3_], [X_1_, W_0_], [K_0_, K_1_], [W_0_, K_0_], [Γ_0_, L_0_], [Γ_0_, L_2_]] that is applied for both spin channels and visually presented in Figure S2. The comparison of reference and optimized band structures are presented in Figure 7. Details of the optimized parameters are provided in the Table S2. To evaluate parameter transferability, we applied the optimized DFTB parameter set to bcc and hexagonal *P*6_3_/*mmc* nickel polymorphs. The electronic band structure comparison in Figure 8 shows excellent agreement with DFT reference calculations, both in terms of band structure features and topology, for both spin channels in the validating structures.

**Figure 7:**
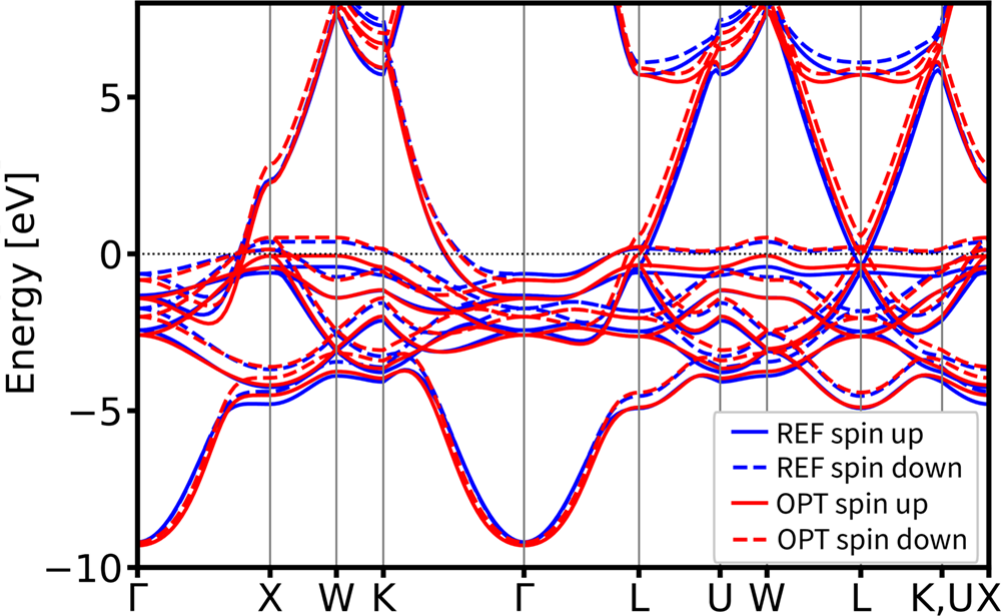
DSKO-optimized band structure (red lines) vs its DFT-calculated reference band structure for the equilibrium geometry of simple cubic Ni (mp-23 from the MP database).

**Figure 8:**
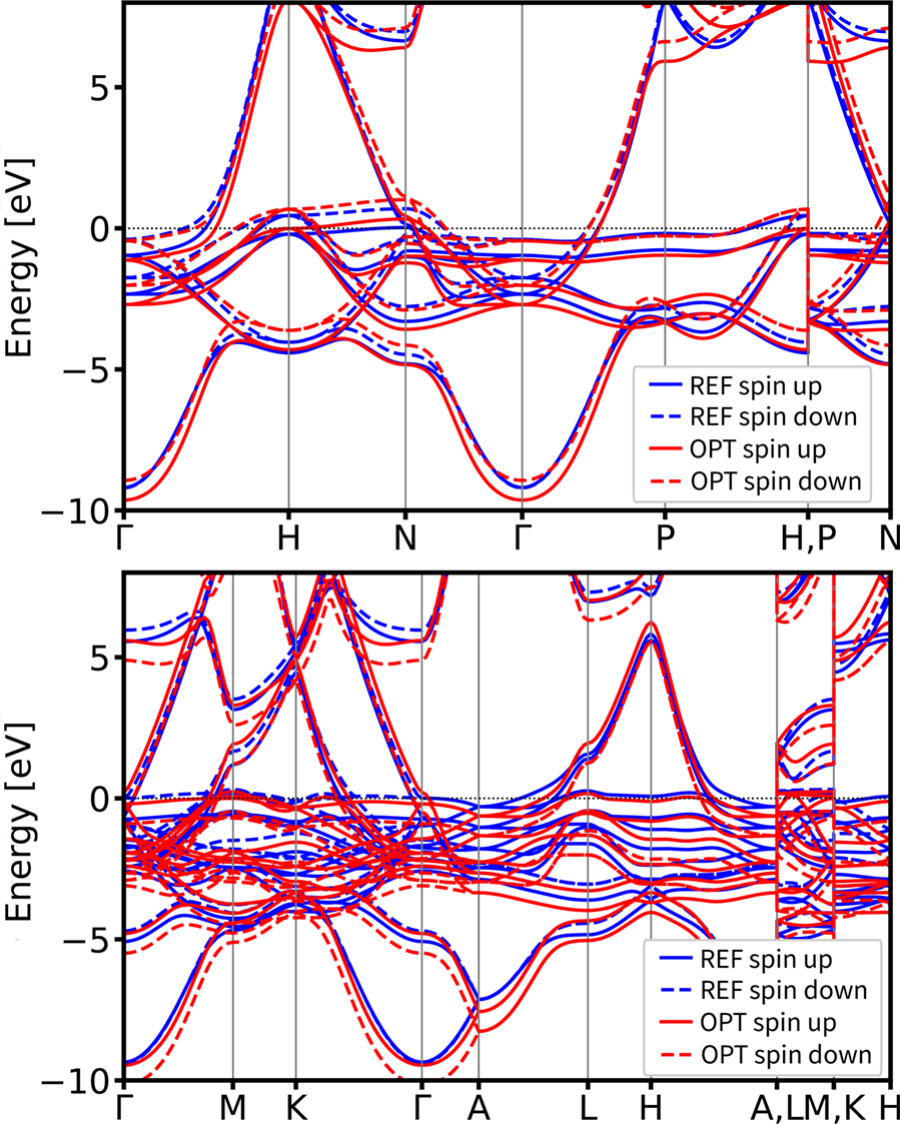
Validation of the band structures of body-centered cubic (bcc) (mp-1008728 from MP database) (top) and hexagonal *P*6_3_/*mmc* (mp-10257 from MP database) (bottom) polymorphs of Ni. All bands are aligned with respect to the DFT Fermi level.

### 4.3 Silicon

The parametrization of silicon involved optimizing shell-resolved wave function confinement and density confinement (both confinements have Woods–Saxon form) using density superposition, without any tuning of onsite energies and Hubbard *U* values, and with an extended basis set of [3s, 3p, 3d] orbitals for Si. The inclusion of the 3d orbital is crucial to correctly reproduce the indirect band gap. As a reference, we have chosen two structures—equilibrium diamond Si (mp-27 from MP database) and its compressed form with a compression factor of 0.9. To evaluate parameter transferability, we applied the optimized DFTB parameter set to lonsdaleite (hexagonal diamond, mp-165 from the MP database) and tetragonal (β-Sn form, mp-92 from the MP database) silicon polymorphs. Here we present two cases—BS and BS + DOS optimizations, while the case of exclusive DOS optimization is presented and discussed in the Supporting Information.

#### 4.3.1 Band Structure Optimization

The fitting procedure targeted the best representation of both band structures, with equal weighting in the objective function. Aligning to the description of the DM in Section 3.3.1, for both input structures we took all paired distances in the range of 3 bands below and 1 band above the Fermi level for {Γ, W, K, L, X, U} special points, as shown in Figure S3. Provided that the 3d orbital is included, the band structure of diamond silicon does not present any particularly problematic point. Therefore, one can write the corresponding input in the simplified point-by-point way as discussed in Section 3.3.1.

The resulting optimized DFTB parameter set successfully reproduces the electronic properties of both structures with high accuracy (see Figure 9). The comparison of the electronic band structure demonstrates strong agreement with the PBE-DFT reference calculation results, especially near the valence band maximum and conduction band minimum. Although it is well-established that PBE-DFT tends to underestimate band gaps, our approach reliably reproduces the reference band structure, even if the reference itself is not perfect. Importantly, any electronic parametrization protocol can target any higher-level DFT approach that surpasses PBE-DFT in accuracy.

**Figure 9:**
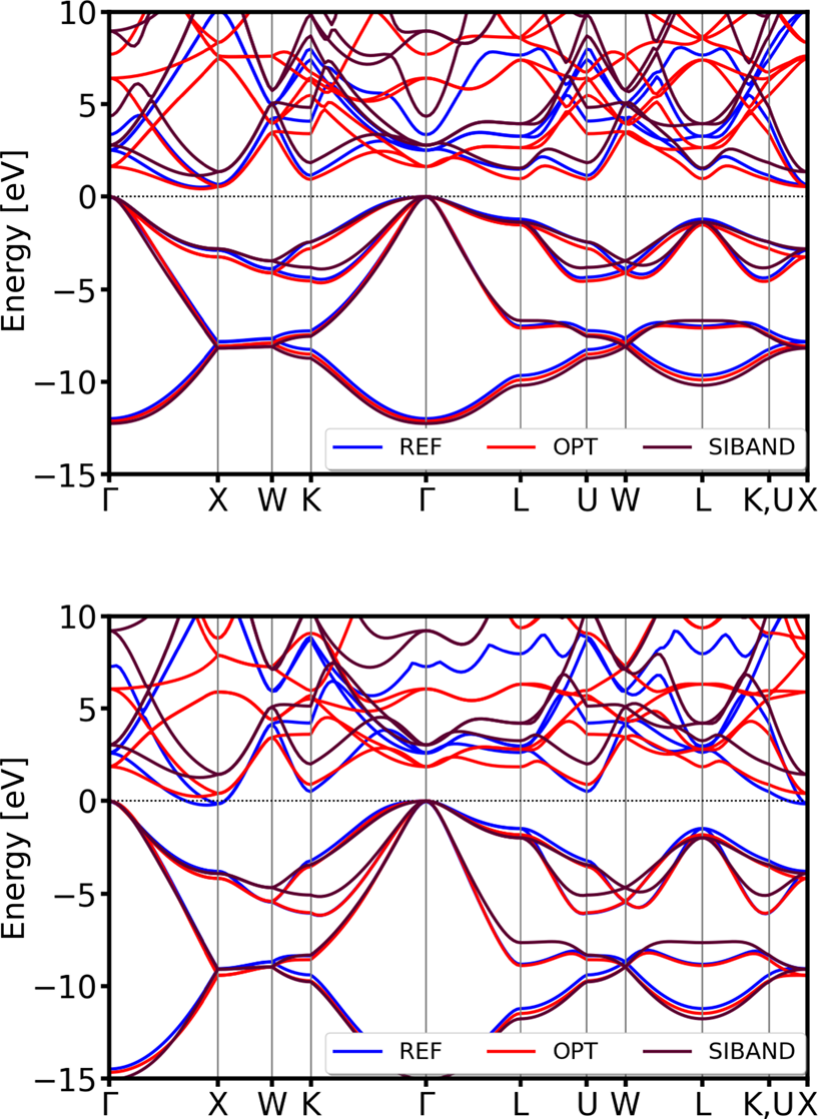
Comparison of the DSKO-optimized (OPT) vs reference (REF) vs obtained by using siband-1-1 DFTB parameters^98,99^ (SIBAND) band structures for the equilibrium geometry (top) and a compressed geometry with a compression factor 0.9 (bottom) of diamond Si. All bands are aligned with respect to the DFT Fermi level.

For both lonsdaleite and tetragonal (β-Sn form) silicon polymorphs, the obtained DFTB parameters accurately maintain the correct band topology compared to the PBE-DFT reference calculation results (see Figure 10). Additionally, these DFTB parameters have been visually compared to the band structures obtained with the siband-1-1 DFTB parameters.^98,99^ It should be noted that the siband-1-1 set was fitted primarily to experimental data (particularly onsite energies); therefore, this comparison is intended only to benchmark our PBE-fitted parameters against an existing parameter set, and we do not emphasize any superiority of the obtained DFTB parameters with respect to the true experimental band structure or to the band structures produced by siband-1-1.

**Figure 10:**
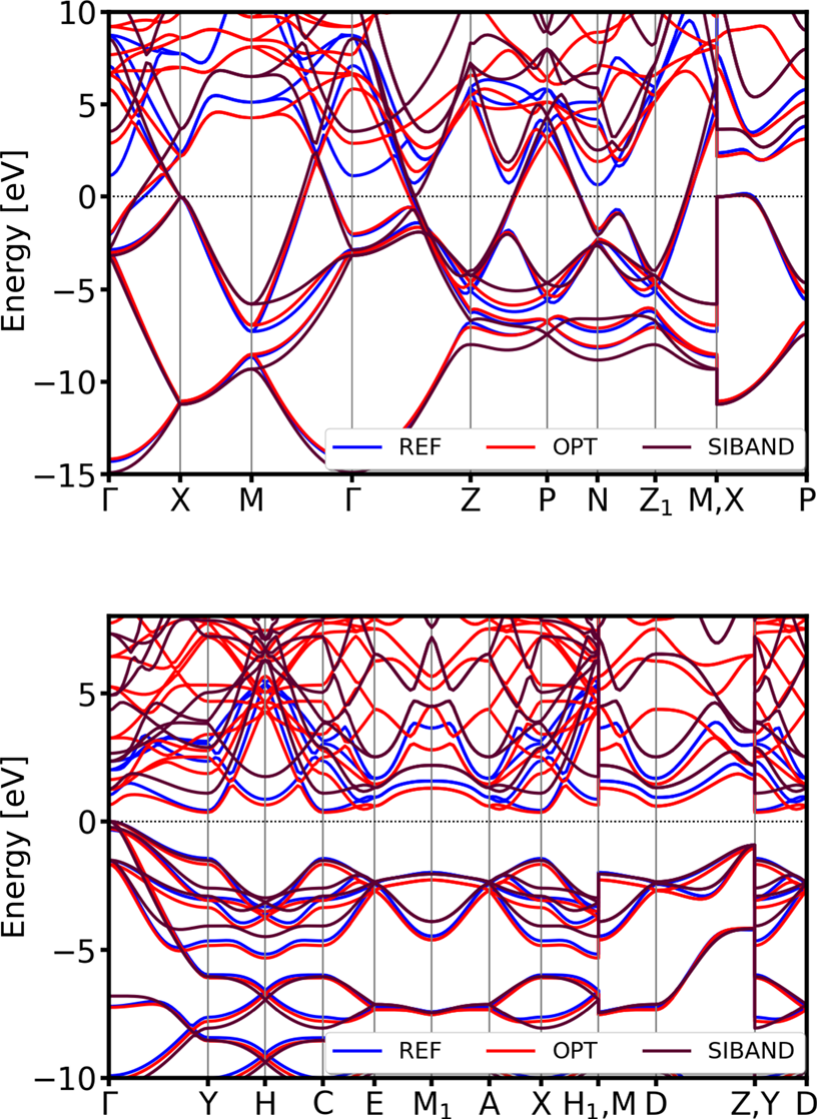
Validation of the band structures of tetragonal (β-Sn) (top) and lonsdaleite (hexagonal diamond) (bottom) polymorphs of Si. All bands are aligned with respect to the DFT Fermi level.

#### 4.3.2 Band Structure and DOS Optimization

The fitting procedure targeted the best representation of both band structures and total DOS, with the objective function weighted equally for BS and DOS for both input structures. The decoding mask for both input structures was the same as in the previous pure BS optimization. The resulting optimized parameter set successfully reproduces the electronic properties of both structures with high accuracy (see Figure 11).

**Figure 11:**
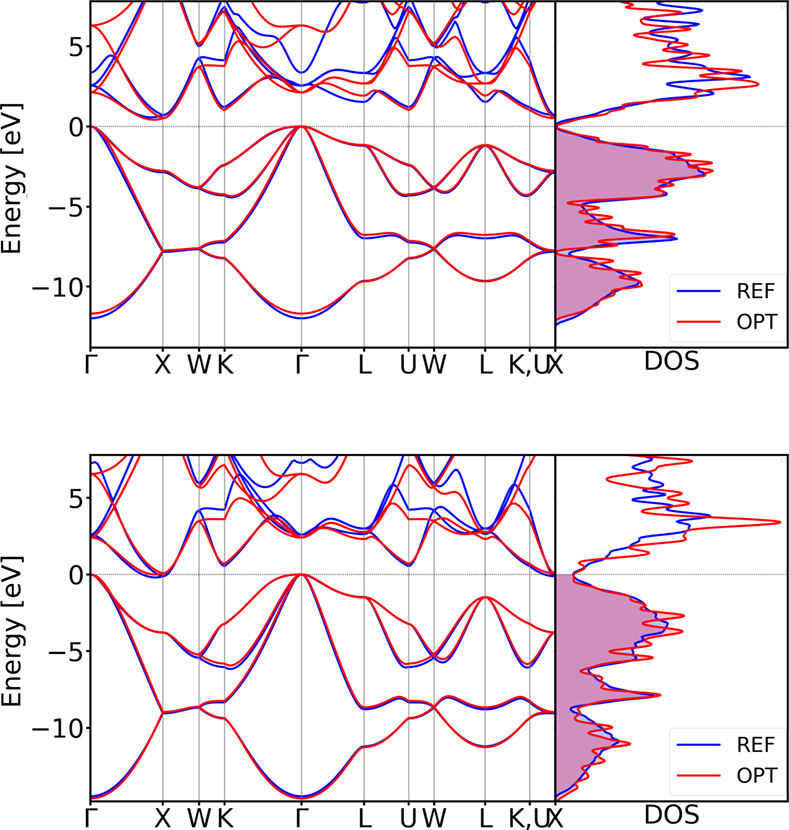
DSKO-optimized band structures and total DOS for the equilibrium geometry (top) and a compressed geometry with a compression factor 0.9 (bottom) of diamond Si. All bands and DOSs are aligned with respect to the DFT Fermi level.

Again, the comparison of both band structure and total DOS shows excellent agreement with the PBE-DFT reference calculation results. For both lonsdaleite and tetragonal (β-Sn form) silicon polymorphs, the obtained parameters accurately maintain the correct band topology compared to the DFT results (see Figure 12).

**Figure 12:**
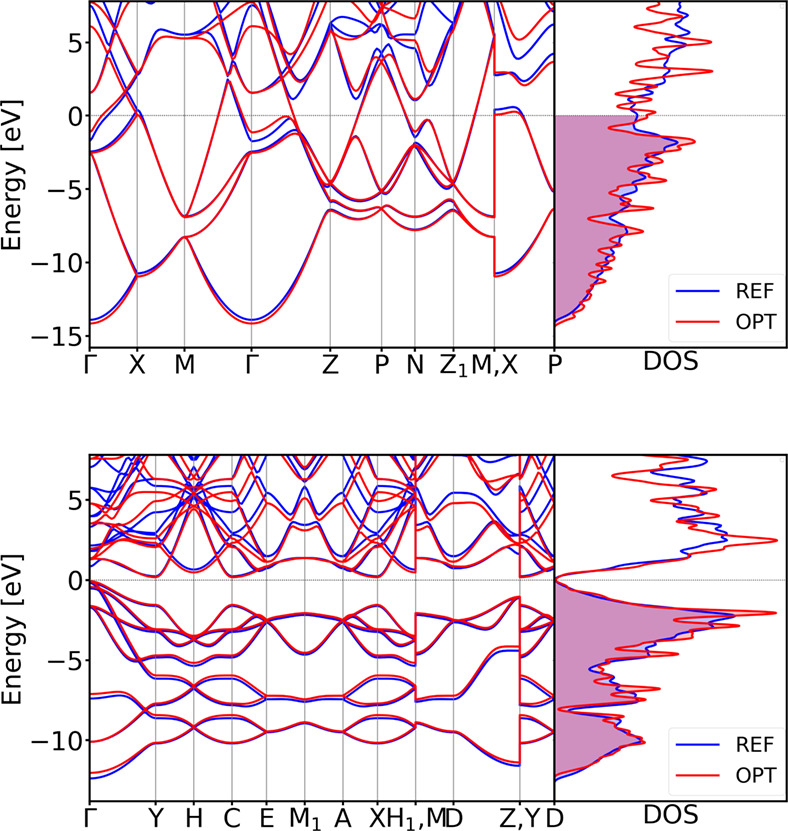
Validation of the band structures of tetragonal (β-Sn) (top) and lonsdaleite (hexagonal diamond) (bottom) polymorphs of Si. All bands are aligned with respect to the DFT Fermi level.

The joint optimization of both BS and DOS yields a well-balanced parametrization. For diamond silicon, BS-only optimization achieves excellent performance for the equilibrium structure (see Figure 10), while joint BS + DOS optimization introduces a modest trade-off in equilibrium BS fidelity but enhances accuracy for compressed structures. This trade-off demonstrates that, while the BS fidelity decreases marginally, the overall electronic structure description becomes more robust. In the case of silicon, a relatively simple system, this minor compromise in BS accuracy is justified by the substantial gain in DOS agreement. Consequently, joint or DOS-focused optimization becomes particularly advantageous for materials with highly folded, “spaghetti-like” band structures, as encountered in crystals with large unit cells, where matching the DOS is often more meaningful than reproducing individual band dispersions. These findings align with the methodological rationale described earlier and underscore the flexibility of the optimization approach (see Figure 12).

Details of the optimized confinement parameters, eigenenergies, and Hubbard values for both BS-only and BS + DOS calculations of Si are provided in the Tables S3 and S4, respectively. Both results show the code’s ability to effectively describe electronic properties across multiple silicon allotropes without requiring separate parametrizations.

### 4.4 Silica

Finally, we present results for a two-element parametrization targeting the band structures of the Si–O system. As a reference, cubic SiO_2_ (mp-10064 from the MP database) was chosen. The DM consists of the following pairs of points: [[Γ_0_, Γ_1_], [X_0_, X_1_], [X_0_, X_2_], [X_1_, K_0_], [K_0_, K_1_], [Γ_1_, X_2_], [X_1_, L_0_], [W_1_, W_2_], [K_0_, K_2_], [L_0_, L_1_], [U_0_, U_1_], [K_–1_, K_1_], [L_–5_, X_–5_], [W_–5_, Γ_1_], [L_1_, L_2_], [X_1_, X_2_], [X_0_, X_3_], [Γ_0_, Γ_2_]]. This DM mainly incorporates points near the Fermi level, along with some from lower bands. A visual representation is provided in Figure S4. The optimization was performed using potential superposition, within the search space of shell-resolved wave function confinement and density confinement parameters (both with Woods–Saxon form), excluding onsite energies and Hubbard *U* values. An extended basis set of [3s, 3p, 3d] orbitals was used for Si, and [2s, 2p] for O.

The optimized DFTB parameters accurately reproduce the electronic structure of cubic SiO_2_, as illustrated in Figure 13. A comparison of the band structures reveals a strong correlation with the PBE-DFT reference calculations, particularly in the vicinity of the valence band maximum and conduction band minimum. Furthermore, the DFTB parameters faithfully capture the indirect band gap presented in the reference band structure. Details of the optimized parameters are provided in the Table S5.

**Figure 13:**
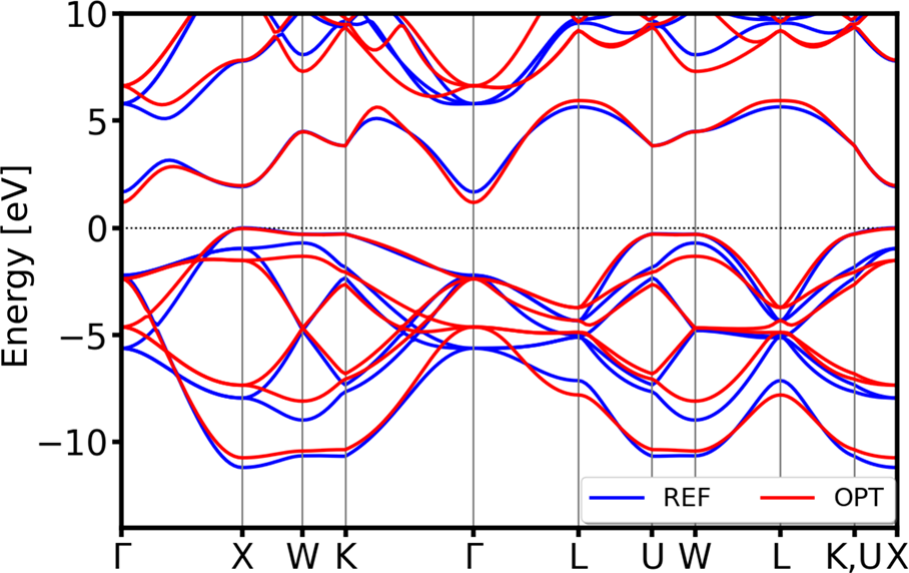
Comparison of the DSKO-optimized (OPT) vs reference (REF) band structure for the equilibrium geometry of cubic SiO_2_ (mp-10064 from the MP database). All bands are aligned with respect to the DFT Fermi level.

Assessing the transferability of the optimized DFTB parameters, we note that they also accurately reproduce orthorhombic *Ibam* SiO_2_ (mp-1071820 from MP database), capturing most of the features and topology of the band structure (see Figure 14). In contrast, the band structure of diamond Si is slightly worse than the one obtained from the DFTB parameter optimization targeting pure Si directly (see above). This outcome illustrates how introducing additional chemical species and targeting multicomponent systems often requires a compromise in DFTB parametrization.

**Figure 14:**
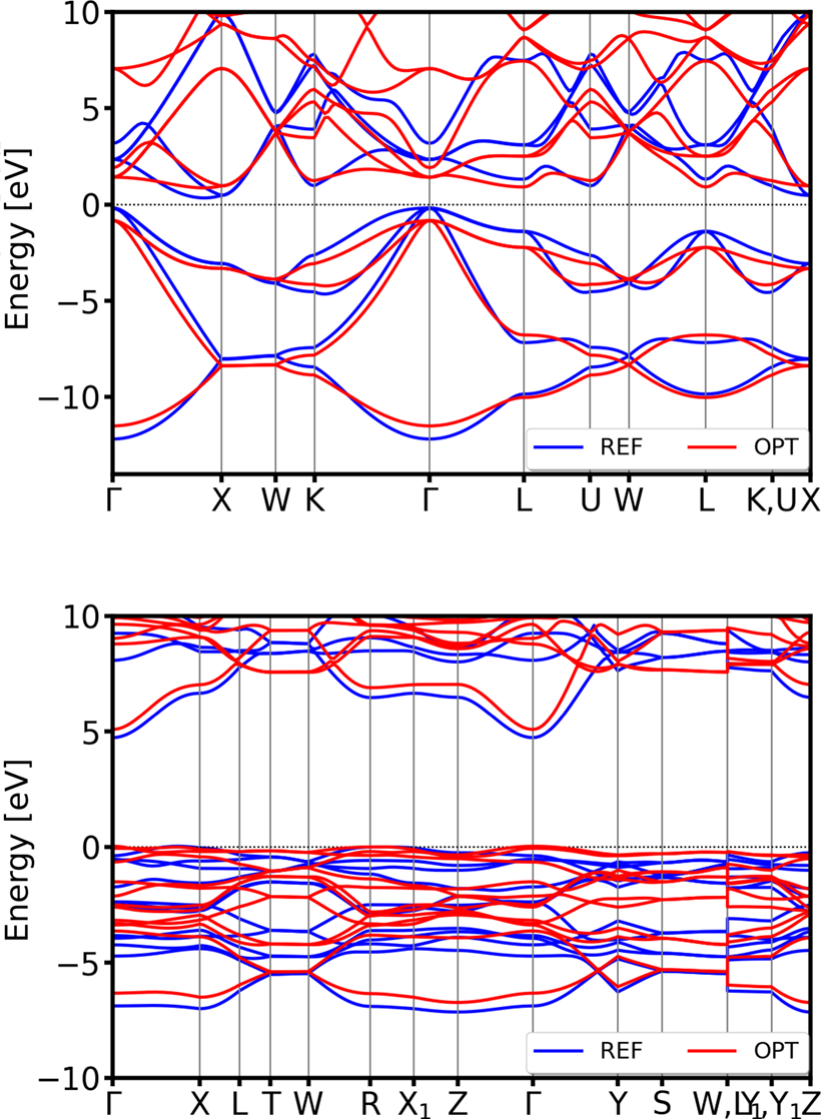
Validation of the transferability of the Si–O DFTB parameter set: band structures of diamond Si (top) and orthorhombic *Ibam* SiO_2_ (bottom). All bands are aligned with respect to the DFT Fermi level.

## 5 Outlook

DFTB parametrization faces a persistent trade-off between specialization and transferability, driving the community toward adaptive workflows that integrate ML with quantum mechanical benchmarking. As ML methods increasingly address numerical challenges such as the generation of high-quality repulsive potentials, the possible imperfections of the approximate electronic structure in DFTB are more than ever exposed and currently in the spotlight. While automated optimization pipelines show promise in addressing reproducibility challenges, several limitations must be addressed. These include systematic errors from the minimal basis set in systems with significant orbital hybridization, angular resolution constraints due to spherical charge density assumptions, and transferability limitations in element-specific parametrization, like in the above case of silicon in diverse environments. Recent advances also highlight the potential of extended basis sets to improve the description of conduction bands, offering a promising avenue to address deficiencies in electronic excitation and band gap predictions.^17,51^ Furthermore, the development and fitting of extended DFTB functionals—such as hybrid or +*U* corrections—represent key directions for enhancing the accuracy and applicability of DFTB to strongly correlated systems. Recent works have demonstrated that long-range corrected (hybrid-like) DFTB and on-site Hubbard *U* extensions can improve the description of charge-transfer excitations and localized d/f electrons, respectively.^17,28,51^

DSKO seeks to mitigate some of these challenges through three key innovations that facilitate precise control of the possible subtleties to address in the electronic parametrization: system-specific parameter generation using global optimization algorithms, dynamic basis set augmentation for targeted bonding environments, and flexible loss function construction. The code is designed to let practitioners explicitly tune the accuracy–transferability balance, i.e., to target either highly system-specific or highly transferable parameter sets within the same framework. For multicomponent chemistries, we envision DSKO being used in a hierarchical fashion: pristine and binary systems are parametrized first, and higher-order mixtures are only treated in a subsequent refinement step that exploits the reference-parameter loss term to constrain the search in parameter space. In its present form, the framework is therefore aimed at delivering accurate and internally consistent parameter sets for well-defined training pools, rather than a single “universal” parametrization that would be optimal across all possible chemistries. System-specific examples in Section 4 highlight the importance of a strategic selection of reference systems and careful loss function design, so rigorous validation of the generated DFTB parameters for each application domain is essential. Currently, DSKO provides functionality for electronic parametrization within the DFTB2 framework only; inclusion of Hubbard parameter derivatives d*U* would extend the parametrization workflow to DFTB3. Finally, as DSKO only addresses electronic parametrization, while energetic accuracy is addressed in a subsequent step using established fitting procedures for repulsive potentials, future development will aim to streamline the two phases of the divide-and-conquer approach within a single software package.

## 6 Conclusions

In this tutorial-style article, we presented our DSKO framework for electronic DFTB parametrization, which exemplifies a divide-and-conquer paradigm by combining physical rigor with global optimization. The workflow employs a flexible loss function that integrates band structure and density of states (DOS) metrics to accurately represent electronic properties. Such flexibility enables practitioners to deliberately adjust the emphasis between accuracy and transferability, yielding either tightly tailored, system-specific parameter sets or broadly applicable parameters suitable across diverse systems. Testing on monatomic (Li, Ni, Si) and binary (SiO_2_) systems demonstrates the approach’s versatility in generating parameters that reproduce DFT electronic properties across diverse crystal environments. The successful validation of the optimized parameters on different polymorphs highlights the potential for achieving improved system-specific (with respect to the existing ones) parametrizations. Looking ahead, integrating the electronic workflow with standardized repulsive fitting (e.g., GPrep^64^), extending to DFTB3 (including Hubbard derivatives), and scaling automated searches and active-learning loops will pave the way for better performance for complex catalytic, energy-storage, and environmental systems. To support adoption, we release code and examples for transparent reuse and benchmarking across materials classes.

## Supplementary Material



## Data Availability

The DSKO code for parametrizing DFTB parameter sets can be found at https://gitlab.mpcdf.mpg.de/fhi-theory/DSKO. For practical use, an interactive web-based companion tool is provided to assist in constructing decoding masks by visualizing reference band structures and enabling the interactive selection of special *k*-points. The tool is available at https://dsko-interactive.onrender.com/viewer and generates decoding masks compatible with the DSKO input format.

## Notes

Computational details, including DFT reference setups, DFTB input parameters, and optimizer configurations for all systems (Li, Ni, Si, and SiO_2_); explicit definitions and illustrative examples of decoding masks used in the optimization workflow for all systems (Li, Ni, Si, and SiO_2_); and tabulated optimized confinement parameters, onsite energies, and Hubbard parameters for all systems (Li, Ni, Si, and SiO_2_) (PDF)
