# Supplementary material for: DSKO: Dancing through DFTB Parametrization
Source: J Chem Theory Comput. 2026 May 1;22(9):4379–93. doi: 10.1021/acs.jctc.6c00121 (PMC13173504; doi:10.1021/acs.jctc.6c00121)
Supplement: Supplementary file 1 [file ct6c00121_si_001.pdf]

Supporting information: DSKO: dancing through DFTB parameterization

Artem Samtsevych,*,† Yihua Song,† Tammo van der Heide,‡ Balint Aradi,¶ Ben Hourahine,§ Reinhard J. Maurer,‖,⊥ Karsten Reuter,† Christoph Scheurer,† and Chiara Panosetti*,†

†*Fritz Haber Institute of the Max Planck Society, 14195 Berlin, Germany*
‡*Bremen Center for Computational Materials Science, Am Fallturm 1, 28359 Bremen, Germany*
¶*Bremen Center for Computational Materials Science, University of Bremen, 28359 Bremen, Germany*
§*Department of Physics, SUPA, University of Strathclyde, John Anderson Building, 107 Rottenrow, Glasgow G4 0NG, United Kingdom*
‖*Department of Chemistry, University of Warwick, Gibbet Hill Road, Coventry CV4 7AL, United Kingdom*
⊥*Department of Physics, University of Warwick, Gibbet Hill Road, Coventry CV4 7AL, United Kingdom*

E-mail: samtsevych@fhi.mpg.de; panosetti@fhi.mpg.de

Contents

1 Computational Details **S1**
1.1 Decoding masks for the input structures S2
1.2 Resulting parameters from DSKO optimizations S4

References **S5**

1 Computational Details

We employed the Atomic Simulation Environment (ASE)[S1] for both pre- and post-processing, including band structure visualization, complemented by the `matplotlib` Python package[S2] for the plotting. Reference band structures and total DOSs were computed with Quantum Espresso[S3,S4] using the Perdew-Burke-Ernzerhof (PBE) functional,[S5] with plane-wave cutoffs of 60 Ry for wave functions and 600 Ry for charge density. We adopted a uniform Γ-centered k-point mesh with a resolution of 0.05 Å^{-1} and applied Gaussian smearing of 0.2 eV for DOS calculations. All DFTB calculations were performed with `DFTB+` (version 24.1)[S6,S7] in shell-resolved SCC mode, using the same k-point density and band path as the DFT reference calculations. In these calculations, we set the electronic temperature to 0.0001 Hartree (∼31.7 K) and enforced an SCC convergence threshold of 10^{-8} electrons. For nickel, we used spin-polarized SCC mode with collinear spin polarization. Initial equilibrium geometries were sourced from the Materials Project (MP) database.[S8] The Particle Swarm Optimization engine control hyperparameters (to regulate the trade-off between exploration and exploitation) were set to $\{c_1 = 0.5, c_2 = 0.3, w = 0.9\}$ for all calculations.

We determined the onsite energies ϵ_μ and Hubbard parameters U_μ using the PBE exchange-correlation functional[S5] as imple-

mented in the Libxc library.[S9] These values were then used as fixed inputs in matrix element calculations with the SkProgs code[S10] or tuned where necessary.

DSKO is fast and efficient; the dominant computational cost arises from generating two-center integrals for the Slater–Koster tables. For each test system, we ran DSKO on a single Linux cluster node equipped with AMD EPYC Genoa 9554 CPUs (128 cores) and 512 GB of memory. A full optimization (400 iterations with a swarm of 128 particles) completed in approximately 3–4 hours of wall-clock time, corresponding to about 500 CPU-hours (128 cores × 3–4 hours).

1.1 Decoding masks for the input structures

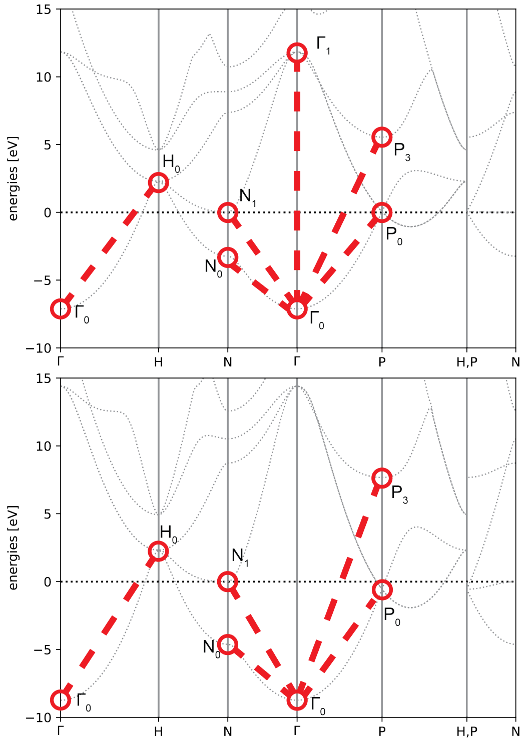

Figure S1: Decoding masks (red lines) on the reference band structures (dotted lines) for body-centered cubic (bcc) lithium (mp − 51 from the MP database) at both equilibrium (top) and a compressed (factor = 0.9, bottom) geometries. For visual clarity, all band structures are aligned to the first conduction band above the Fermi level at the N special point, rather than to the DFT Fermi level. This unconventional alignment is chosen solely to enhance the readability of the plots.

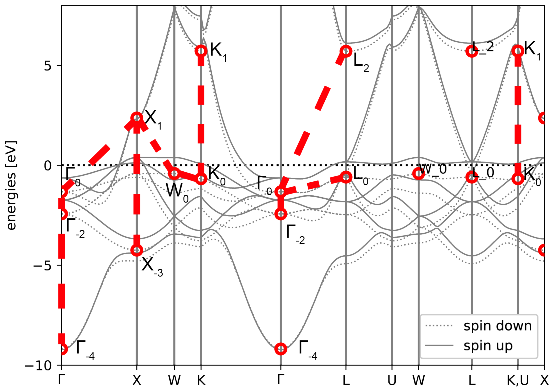

Figure S2: Decoding masks (red lines) are shown on the reference band structures (dotted lines) for simple cubic Ni (mp-23 from the MP database). For clarity, the decoding mask is constructed only for the spin-down channel; for the spin-up channel, the mask consists of the same pairs of points.

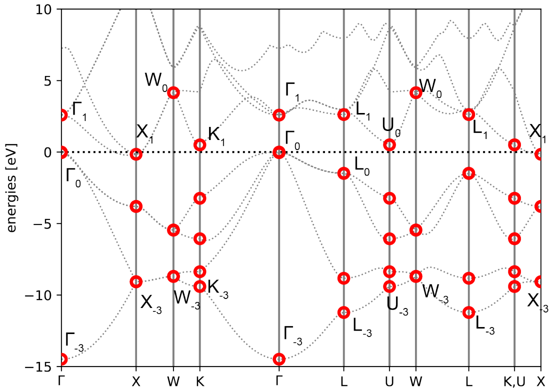

Figure S3: Decoding masks are shown on the reference band structures (dotted lines) for compressed (factor 0.9) of a diamond Si $(mp-27$ from MP database). The decoding mask is constructed as all paired distances in the range of 3 bands below and 1 bands above the Fermi level for $\{\Gamma, W, K, L, X, U\}$ special points. All the band structures are aligned with respect to the DFT Fermi level.

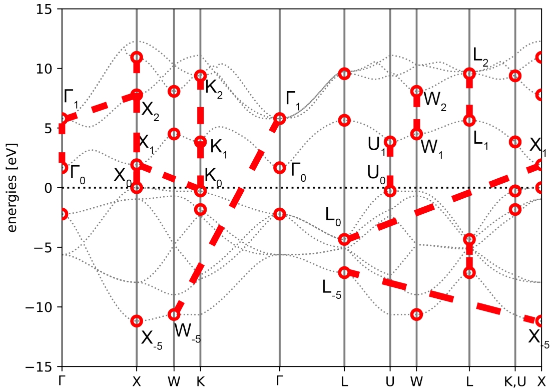

Figure S4: Decoding masks are shown on the reference band structures (dotted lines) for equilibrium geometry of cubic SiO_2 (mp-10064 from the MP database). The DM consists of the following pairs of points: $[[\Gamma_0, \Gamma_1]$, $[X_0, X_1]$, $[X_0, X_2]$, $[X_1, K_0]$, $[K_0, K_1]$, $[\Gamma_1, X_2]$, $[X_1, L_0]$, $[W_1, W_2]$, $[K_0, K_2]$, $[L_0, L_1]$, $[U_0, U_1]$, $[K_{-1}, K_1]$, $[L_{-5}, X_{-5}]$, $[W_{-5}, \Gamma_1]$, $[L_1, L_2]$, $[X_1, X_2]$, $[X_0, X_3]$, $[\Gamma_0, \Gamma_2]]$. All the band structures are aligned with respect to the DFT Fermi level.

1.2 Resulting parameters from DSKO optimizations

Table S1: Resulting parameters for Li optimized using `DSKO`. An extended valence basis set including s and p orbitals is employed. For both wavefunction and density confinement, the Woods–Saxon parameters (r_0, a, and W) are reported. The eigenvalues of the atomic orbitals are also listed, as they were the primary targets of the optimization.

		**Woods–Saxon Parameters**		
		r_0	a	W
**Li**	wf(s)	6.115	57.750	334.960
	wf(p)	6.797	52.500	349.723
	wf(d)	—	—	—
	dens	9.093	78.797	250.833

Eigenvalues: $\varepsilon_s = -0.109$ Ha, $\varepsilon_p = -0.039$ Ha

Table S2: Resulting parameters for Ni optimized using `DSKO`. An extended valence basis set including s, p and d orbitals is employed. For both wavefunction and density confinement, the Woods–Saxon parameters (r_0, a, and W) are reported.

		**Woods–Saxon Parameters**		
		r_0	a	W
**Ni**	wf(s)	3.733	32.894	317.059
	wf(p)	4.783	44.118	210.373
	wf(d)	5.076	48.779	220.665
	dens	5.750	22.734	219.079

Table S3: Resulting parameters for Si optimized using `DSKO`. An extended valence basis set including s, p and d orbitals is employed. For both wavefunction and density confinement, the Woods–Saxon parameters (r_0, a, and W) are reported.

		**Woods–Saxon Parameters**		
		r_0	a	W
**Si**	wf(s)	4.987	81.839	286.122
	wf(p)	5.604	68.517	369.705
	wf(d)	6.399	73.293	457.482
	dens	41.844	62.824	273.594

Table S4: Resulting parameters for Si optimized in BS+DOS mode using `DSKO`. An extended valence basis set including s, p and d orbitals is employed. For both wavefunction and density confinement, the Woods–Saxon parameters (r_0, a, and W) are reported.

		**Woods–Saxon Parameters**		
		r_0	a	W
**Si**	wf(s)	5.028	81.986	286.273
	wf(p)	5.628	68.356	369.675
	wf(d)	6.016	73.202	457.428
	dens	41.051	62.789	273.576

Table S5: Resulting parameters for Si and O optimized using `DSKO`. An extended valence basis set including s, p and d orbitals for Si and s and p orbitals for O is employed. For both wavefunction and density confinement, the Woods–Saxon parameters (r_0, a, and W) are reported.

		**Woods–Saxon Parameters**		
		r_0	a	W
**Si**	wf(s)	3.894	28.247	448.193
	wf(p)	4.038	60.781	350.784
	wf(d)	5.434	71.694	304.970
	dens	16.871	46.313	305.211
**O**	wf(s)	2.441	13.881	359.852
	wf(p)	4.151	27.489	310.523
	wf(d)	—	—	—
	dens	27.526	47.108	244.787

References

[S1] Larsen, A.; Mortensen, J.; Blomqvist, J.; Castelli, I.; Christensen, R.; Dułak, M.; Friis, J.; Groves, M.; Hammer, B.; Hargus, C.; Hermes, E.; Jennings, P.; Jensen, P.; Kermode, J.; Kitchin, J.; Kolsbjerg, E.; Kubal, J.; Kaasbjerg, K.; Lysgaard, S.; Maronsson, J.; Maxson, T.; Olsen, T.; Pastewka, L.; Peterson, A.; Rostgaard, C.; Schiøtz, J.; Schütt, O.; Strange, M.; Thygesen, K.; Vegge, T.; Vilhelmsen, L.; Walter, M.; Zeng, Z.; Jacobsen, K. The atomic simulation environment—a Python library for working with atoms. *J. Phys.: Condens. Matter* **2017**, *29*, 273002.

[S2] Hunter, J. Matplotlib: A 2D graphics environment. *Computing in science & engineering* **2007**, *9*, 90–95.

[S3] Giannozzi, P.; Baroni, S.; Bonini, N.; Calandra, M.; Car, R.; Cavazzoni, C.; Ceresoli, D.; Chiarotti, G.; Cococcioni, M.; Dabo, I.; Dal Corso, A.; de Gironcoli, S.; Fabris, S.; Fratesi, G.; Gebauer, R.; Gerstmann, U.; Gougoussis, C.; Kokalj, A.; Lazzeri, M.; Martin-Samos, L.; Marzari, N.; Mauri, F.; Mazzarello, R.; Paolini, S.; Pasquarello, A.; Paulatto, L.; Sbraccia, C.; Scandolo, S.; Sclauzero, G.; Seitsonen, A.; Smogunov, A.; Umari, P.; Wentzcovitch, R. QUANTUM ESPRESSO: a modular and open-source software project for quantum simulations of materials. *J. Phys.: Condens. Matter* **2009**, *21*, 395502 (19pp).

[S4] Giannozzi, P.; Andreussi, O.; Brumme, T.; Bunau, O.; Nardelli, M.; Calandra, M.; Car, R.; Cavazzoni, C.; Ceresoli, D.; Cococcioni, M.; Colonna, N.; Carnimeo, I.; Corso, A.; de Gironcoli, S.; Delugas, P.; Jr, R.; Ferretti, A.; Floris, A.; Fratesi, G.; Fugallo, G.; Gebauer, R.; Gerstmann, U.; Giustino, F.; Gorni, T.; Jia, J.; Kawamura, M.; Ko, H.-Y.; Kokalj, A.; Küçükbenli, E.; Lazzeri, M.; Marsili, M.; Marzari, N.; Mauri, F.; Nguyen, N.; Nguyen, H.-V.; Otero-de-la Roza, A.; Paulatto, L.; Poncé, S.; Rocca, D.; Sabatini, R.; Santra, B.; Schlipf, M.; Seitsonen, A.; Smogunov, A.; Timrov, I.; Thonhauser, T.; Umari, P.; Vast, N.; Wu, X.; Baroni, S. Advanced capabilities for materials modelling with QUANTUM ESPRESSO. *J. Phys.: Condens. Matter* **2017**, *29*, 465901.

[S5] Perdew, J.; Burke, K.; Ernzerhof, M. Generalized gradient approximation made simple. *Phys Rev Lett* **1996**, *77*, 3865.

[S6] Aradi, B.; Hourahine, B.; Frauenheim, T. DFTB+, a sparse matrix-based implementation of the DFTB method. *J. Phys. Chem. A* **2007**, *111*, 5678–5684.

[S7] Hourahine, B.; Aradi, B.; Blum, V.; Bonafé, F.; Buccheri, A.; Camacho, C.; Cevallos, C.; Deshaye, M.; Dumitrică, T.; Dominguez, A.; Ehlert, S.; Elstner, M.; van der Heide, T.; Hermann, J.; Irle, S.; Kranz, J.; Köhler, C.; Kowalczyk, T.; Kubař, T.; Lee, I.; Lutsker, V.; Maurer, R.; Min, S.; Mitchell, I.; Negre, C.; Niehaus, T.; Niklasson, A.; Page, A.; Pecchia, A.; Penazzi, G.; Persson, M.; Řezáč, J.; Sánchez, C.; Sternberg, M.; Stöhr, M.; Stuckenberg, F.; Tkatchenko, A.; Yu, V.; Frauenheim, T. DFTB+, a software package for efficient approximate density functional theory based atomistic simulations. *J. Chem. Phys.* **2020**, *152*, 124101.

[S8] Jain, A.; Ong, S.; Hautier, G.; Chen, W.; Richards, W.; Dacek, S.; Cholia, S.; Gunter, D.; Skinner, D.; Ceder, G.; others Commentary: The Materials Project: A materials genome approach to accelerating materials innovation. *APL Mater.* **2013**, *1*.

[S9] Lehtola, S.; Steigemann, C.; Oliveira, M.; Marques, M. Recent developments in

libxc—A comprehensive library of functionals for density functional theory. *SoftwareX* **2018**, *7*, 1–5.

[S10] van der Heide, T.; Aradi, B.; Hourahine, B.; DFTB+ Project SkProgs: Basic programs for generating Slater–Koster files for the DFTB method. 2024; `https://github.com/dftbplus/skprogs`.
